# A collagen hydrogel–based 3D Heparg model for enhanced hepatocyte function and assessment of cholestatic drug-induced liver injury

**DOI:** 10.1007/s00204-026-04324-z

**Published:** 2026-03-10

**Authors:** Enrique Timor-López, Laia Tolosa, M. Teresa Donato

**Affiliations:** 1 Experimental Hepatology Unit, Health Research Institute La Fe (IISLAFE), 46026 Valencia, Spain; 2 Faculty of Medicine and Dentistry, Department of Biochemistry and Molecular Biology, University of Valencia, 46010 Valencia, Spain; 3 Biomedical Research Networking Centre On Bioengineering, Biomaterials and Nanomedicine (CIBER-BBN), Carlos III Health Institute, 46022 Valencia, Spain; 4 Biomedical Research Networking Center in Hepatic and Digestive Diseases (CIBEREHD), Carlos III Health Institute, 28029 Madrid, Spain

**Keywords:** HepaRG cells, 3D culture, Collagen, DILI, Drug-induced cholestasis

## Abstract

Drug-induced liver injury (DILI) is a major clinical and regulatory challenge, with cholestasis representing a frequent mechanism of hepatotoxicity. Conventional two-dimensional (2D) hepatocyte cultures often fail to predict such effects, underscoring the need for advanced models that better replicate liver architecture and function. In this study, a three-dimensional (3D) model was developed by encapsulating HepaRG cells within type I collagen hydrogels to enhance functional maturation and reduce differentiation time compared to standard 2D cultures. The 3D HepaRG model maintained long-term viability and exhibited improved hepatic functions, including urea and albumin production, xenobiotic metabolism, and antioxidant defense, particularly after 14 days of culture with a reduced amount of DMSO (1%) compared to the standard 2D protocol. When incubated with cholestatic antibiotics, such as cloxacillin, flucloxacillin, and nafcillin, the 3D model reproduced cholestatic DILI features, showing increased cytotoxicity in the presence of bile acids and after prolonged exposure. Transcriptomic profiling revealed dose-dependent modulation of genes involved in bile acid homeostasis. A more in-depth analysis of cloxacillin-toxicity using RNA-seq revealed an altered expression of genes involved in bile acid metabolism, oxidative stress, and endoplasmic reticulum stress. Functionally, cloxacillin induced oxidative stress, mitochondrial dysfunction, and activation of apoptotic signaling pathways. Overall, the collagen hydrogel–based 3D HepaRG model provided a robust and physiologically relevant platform for investigating antibiotic-induced cholestatic DILI and its underlying mechanisms, offering improved predictivity over traditional 2D systems and valuable potential for preclinical drug safety assessment.

**Supplementary Information:**

The online version contains supplementary material available at 10.1007/s00204-026-04324-z.

## Introduction

Drug-induced liver injury (DILI) is a multifactorial potentially life-threatening adverse reaction to prescribed drugs and other chemicals (Fernandez-Checa et al. 2021). DILI is both a cause of drug attrition in developmental phases and a major cause of drug withdrawal after commercialization, which poses a challenge for the pharmaceutical industry. Approximately, the 25% of drugs withdrawals (Onakpoya et al. 2016) and 20% of black box warnings (Lasser et al. 2002) of prescription medications are due to DILI. The variability of the clinical symptoms, severity, and consequences of DILI is significant because a vast array of clinical manifestations is indicative of toxic liver damage, ranging from asymptomatic and temporary increases in serum levels of liver enzymes to acute liver failure. From a clinical point of view, hepatotoxicity patterns are classified as hepatocellular, cholestatic, or mixed, using consensus criteria based on relative ratios of alanine aminotransferase and alkaline phosphatase activity levels (Aithal et al. 2011). According to Oorts et al. (2015), cholestatic injury actually accounts for up to 50% of reported DILI cases (Oorts et al. 2015). About 73% of all cholestatic DILI cases are caused by single prescription drugs, primarily antibiotics, antifungals, immunomodulators, antipsychotics, hypoglycemic drugs and steroids (Gijbels et al. 2019).

An ideal scenario would be to remove drugs with an elevated risk of causing DILI early in the drug development process. Until now, preclinical drug safety assessments have still heavily relied on animal experiments, but they have significant limitations due to ethical concerns and the substantial differences in drug metabolism and pharmacokinetics between experimental animals and humans (O'Brien et al. 2004). Although in vitro models have many limitations, current legislation strongly encourages the use of new alternative methodologies to predict DILI because it implies reduced time and costs as well as replacing, reducing and refining animal use (3 Rs) in safety and efficacy evaluations. Thus, the development of human cell-based models for relevant DILI predictions has become a major challenge in preclinical assessment.

In the last decade, the human HepaRG cell line has been increasingly used for drug toxicity studies (Franzosa et al. 2021; Hammour et al. 2022; Thienpont et al. 2023; Tolosa et al. 2016a; Tomida et al. 2015). To acquire an optimal hepatocyte-like phenotype, undifferentiated HepaRG cells cultured in conventional 2D monolayers need to be maintained for 14-days under proliferating conditions, followed by additional 14-days in the presence of DMSO (Aninat et al. 2006; Hammour et al. 2022; Sharanek et al. 2015). Both the long-lasting culture period and the relatively high DMSO concentration (around 1.7—2%) required for the differentiation of HepaRG limit their application to toxicity studies. Alternatively, cryopreserved differentiated HepaRG cells can be supplied by the vendor, which could be advantageous; however, these cells have notable limitations, such as being subjected to restricted use policies, not being able to be propagated or subcultured, and their high cost. Recent studies have shown that 3D culture models promote hepatic differentiation of HepaRG cells (Liao et al. 2022; Rose et al. 2022; Wang et al. 2019). 3D cultures are more physiological liver cell models that improve cell–cell and cell–matrix interactions and maintenance of cell polarity.

The objective of this study was to explore the use of HepaRG cells differentiated in 3D cultures based on collagen hydrogels as an in vitro model for hepatotoxicity studies. To this end, a comprehensive characterization of the 3D model was performed by analyzing gene expression profiles and typical liver functions compared to 2D cultures. The 3D model was subsequently applied to the study of DILI, specifically to cholestatic liver damage induced by three beta-lactam antibiotics (cloxacillin, flucloxacillin and nafcillin). The results suggest that the HepaRG 3D model is a valuable system for evaluating the cholestatic potential of new drugs and for further understanding of the hepatotoxicity mechanisms involved.

## Materials and methods

### Materials

Culture media, complements and buffers were purchased from GIBCO (Gibco BRL, Paisley, UK). Foetal bovine serum (FBS), inductors, cytochrome P450 (CYP) substrates and test compounds were acquired from Merck (Darmstadt, Germany). Primers were acquired from ThermoFisher Scientific (Waltham, Massachusetts, USA). Unless otherwise indicated, all other chemicals were purchased from Merck (Darmstadt, Germany).

### Cell culture

Cryopreserved undifferentiated HepaRG cells were obtained from Biopredic International (Rennes, France). For 2D cultures, cells were seeded at a density of 2.8 × 10^4^ cells/cm^2^ and were cultured for 2 weeks in Williams’ E medium supplemented with 10% FBS, 2 mM glutamine, 100 IU/ml penicillin, 100 µg/ml streptomycin, 5 µg/ml insulin and 5 µM hydrocortisone hemisuccinate, according to Sharanek et al. (2015). Then, cells were cultured for another 2-week period in the same medium supplemented with 1.7% DMSO. Medium was replaced every 2—3 days. For 3D cultures, a 2.5 mg/ml collagen solution was prepared immediately before use by mixing a solution of 3 mg/ml type I collagen from rat tail tendon (Corning 11.179.179.001) in 0.2% acetic acid, culture medium and basic an acidic solutions (0.7 M NaOH / 0.26 M NaHCO3 and 1% acetic acid) to adjust pH to 7.4 (10:1:1, v/v/v). HepaRG cells were resuspended in the collagen solution at a density of 1 × 10^6^ cells/ml and this cell preparation was poured into 48-well plates (100 µl/well) and incubated at 37 °C, 5% CO_2_. After 60 min, the gels were polymerized and 300 µl of cultured medium was added to each well. On days 2 and 4, the medium was replaced with medium supplement with 0.5% and 1% DMSO, respectively. Thereafter, medium with 1% DMSO was refreshed every 2—3 days. Supplementary Figure S1 shows the experimental design for 2D and 3D HepaRG cell cultures. Cell viability was monitored in 2D and 3D cultures by incubation with Hoechst 33,342 (HO) (1.5 μg/ml) and propidium iodide (1.5 μg/ml) for 30 min (live/dead assay) and capture of cell images with a Leica CTR4000 microscope (Leica Microsystems, Wetzlar, Germany).

### RNA extraction and transcriptomic analysis

Total RNA was obtained according to the protocol of RNAeasy kit (Qiagen, Madrid, Spain) and its concentration and purity were assessed by measuring A260 and A260/A280 ratio, respectively, in a NanoDrop1000 spectrophotometer (ThermoFisher Scientific, Waltham, Massachusetts, USA). Total RNA (1 µg) was reverse-transcribed and diluted cDNA was amplified using LightCycler DNA Master SYBR Green I (Roche Applied Science, Barcelona, Spain) and the appropriate primers (Supplementary Table S1) using a LightCycler480 Instrument (Roche Applied Science, Barcelona, Spain). HMBS (hydroxymethylbilane synthase), GAPDH (glyceraldehyde 3-phosphate dehydrogenase) and TBP (TATA binding protein) were used as reference housekeeping genes. A reference calibrator cDNA, made from a pool of 18 human livers, was included in each amplification. Based on PCR efficiencies and the cycle thresholds, the relative concentration of the target and reference cDNAs and their ratio were determined.

### Immunofluorescence

Cells were fixed in 4% paraformaldehyde for 30 min at room temperature and washed 3 times with phosphate buffer saline (PBS). After fixing, the samples were immersed in 30% sucrose-Dulbecco’s PBS (DPBS) overnight and then covered with optimal cut temperature embedding matrix. Cryosections (60 μm) were obtained with a cryostat. Permeabilization was performed using 0.5% Triton X-100 in PBS for 30 min at room temperature and blocking was carried out with 3% bovine serum albumin (BSA) in PBS for 2 h at room temperature. Samples were then subsequently incubated with a primary antibody overnight at 4 °C and a secondary antibody for 1 h at room temperature conveniently diluted in PBS-BSA 1% (Supplementary Table S2). Nuclei were counterstained with 2 μg/ml HO for 5 min. Visualization was performed with an inverted fluorescence microscopy Leica DM2500 LED (Leica Microsystems, Wetzlar, Germany). Specificity controls were performed by omitting the primary antibody.

### Hepatic functionality and drug metabolism assessment

The functionality of HepaRG cells in different culture conditions was assessed as previously described in detail (Tolosa et al. 2016b). Urea synthesis and albumin production were measured in culture medium and values were related to total RNA in cell culture and were expressed as ng of urea or albumin/h/μg RNA.

Cytochrome P450 (CYP) and UDP-glucuronosyltransferase (UGT) activities were assayed by incubation of cell cultures for 3 h with a mixture of selective substrates for individual CYP enzymes or UGT1A1 or UGT2B7, respectively. Quantification of the corresponding metabolites was performed by high-performance liquid chromatography coupled with tandem mass spectrometry as previously described (Donato et al. 2010; Lahoz et al. 2007). Activity values were expressed as pmol of metabolite/h/μg RNA.

Glutathione-S-transferase (GST) and glutathione peroxidase (GPX) activities were measured as previously described (Donato et al. 2022, 2015). Glutathione reductase (GSR) activity was determined with commercial kit (Elabscience, Houston, Texas, USA) following the established procedures. All the enzymatic activities were expressed as U (or mU)/μg RNA.

Transport by apical transporter MRP2 was assessed with an assay based on the compound 5(6)-carboxy-2′,7′-dichlorofluorescein diacetate (CDFDA), which is taken up by the hepatocytes, cleaved by intracellular esterases and finally released to the bile canaliculi. As the product is fluorescent, its location can be tracked by fluorescence microscopy. HepaRG cells in 3D culture were washed with Hank’s balanced saline solution, incubated in the same buffer for 10 min at 37 ºC, 5% CO_2_, and then in 5 µM CDFDA for 20 min under the same conditions. Later, the samples were washed three times and immediately photographed in a Leica CTR4000 microscope.

### Selection of drugs and treatments

Three β-lactam antibiotics, cloxacillin (CLO), flucloxacillin (FLU) and nafcillin (NAF), were assessed in the present study. In addition, bosentan (BOS), a model cholestatic compound, and streptomycin (STR), a non-hepatotoxic antibiotic, were used as positive and negative controls, respectively. Detailed information of the drugs is included in Table 1. Stock solutions of the drugs were prepared in DMSO and stored at – 20 ºC until used. Thawed solutions were freshly diluted in the culture medium with or without a mixture of bile acids (BAs) to reach the desired concentrations and the same amount of DMSO was added to control cultures. BA mixture consisted of eight BA at a concentration 100 times higher than that in human plasma (Ogimura et al. 2011; Scherer et al. 2009) (Supplementary Table S3). In all cases, the final DMSO concentration in culture medium was 1.4%. After 14 days in culture, HepaRG cells in 3D culture were exposed during 24 h to several concentrations of the drugs in the presence or absence of a BA mixture. For long-treatment experiments, cells were treated with drugs following a repeated-dose regimen for 3, 7 and 14 days (using two, four or seven-repeated doses, respectively).

**Table 1:** Test compounds

Compound	Type	Category	Tested concentrations (mM)	C_max (µM)_	References
Cloxacillin	β-lactamic antibiotic	Hepatotoxic cholestatic	0–35	184	Mensa et al. (2013)
Flucloxacillin	β-lactamic antibiotic	Hepatotoxic cholestatic	0–35	191	Gardiner et al. (2018)
Nafcillin	β-lactamic antibiotic	Hepatotoxic cholestatic	0–21	116	Feldman et al. (1978)
Bosentan	Pulmonary antihypertensive	Hepatotoxic cholestatic	0–3.5	136	Dingemanse and van Giersbergen (2004)
Streptomycin	Aminoglycoside antibiotic	Not hepatotoxic	0–8	34	Tolosa et al. (2019)

### Hepatotoxicity assessment

The release of intracellular lactate dehydrogenase (LDH) was used as an indicator of cellular damage. After treatments, LDH activity present in culture medium was determined spectrophotometrically using LDH Kit (Spinreact, Gerona, Spain) and the results were expressed as fold over activity values in untreated cells. Cellular ATP content was determined as a marker of cell viability using the CellTiter-Glo® 3D Cell Viability Assay (Promega, Madison, Wisconsin, USA). To this, ATP reagent was added to each well and after 30 min of incubation at room temperature, luminescence from luciferase reaction was recorded with a BioTek Synergy H1 Multimode Reader (Agilent, Santa Clara, California, USA). Viability data were expressed as the percentage of luminescence intensity of cell treated with drugs compared to control (untreated cells). Then, the concentrations causing 10% and 50% reductions in cell viability (IC10 and IC50) were estimated for each compound in the absence or presence of the BA cocktail and these values were used to calculate the cholestatic index (Clx) that compares the cytotoxicity of a drug alone or co-exposed with BAs (Hendriks et al. 2016; Gijbels et al 2020). In our study, Clx was calculated from data of ATP content as the ratio of the IC50 (or IC10) of the compound plus BA mix divided by the IC50 (or IC10) of the compound alone. Compounds with CIx values < 0.8 were considered to have a cholestatic potential (Hendriks et al. 2016).

In order to elucidate the role of p38 in the toxicity induced by CLO, the p38 inhibitor adezmapimod (SB203580 10 µM) was co-incubated with CLO in the presence or absence of the BA mixture.

### High-content screening assay for CLO-induced toxicity

Following treatment with CLO, cells were simultaneously loaded with a combination of fluorescent probes to measure the multiple parameters that were indicative of different mechanisms of cell toxicity (Tolosa et al. 2012). To this, HO (cell number determination), Fluo-4 AM (changes in cytosolic-free calcium concentrations), CellROX Deep Red (ROS production) and MitoSOX Red (production of superoxide by mitochondria) were used at the concentrations indicated in Supplementary Table S4. After incubating with dyes, cells were imaged by the DMi8 microscope (Leica). Dyes were excited and their fluorescence was monitored at the excitation and emission wavelengths with appropriate filter settings. The collected images were analyzed using the ImageJ software, which allows the simultaneous quantification of the subcellular structures stained by different fluorescent probes and by measuring the fluorescence intensity associated with predefined nuclear and cytoplasmic compartments.

### RNA sequencing

Global transcriptome profiling was performed. At least 1 μg of RNA from untreated 3D HepaRG cells and cells treated with different concentrations of CLO in the absence or presence of BAs was extracted as described in previous Sect. (2.3) for analysis by RNA sequencing (RNAseq). To quantify and identify the mRNAs, a total of approximately 15 million reads were generated in each library. Alignment was performed using the STAR tool, using the Homo sapiens GRCh38p13 genome sequence as a reference. Read counts were calculated, eliminating PCR duplicates, and differential gene expression analysis was performed with the support of the Genomics Service (University of Valencia).

Differential expression analysis was conducted at the gene level across all experimental groups using the normalized gene counts generated by RSEM. Pairwise comparisons (e.g., Group A vs Group B) were performed, and results were considered statistically significant based on adjusted p-values (FDR < 0.05) and fold change thresholds. Gene expression levels were estimated based on transcript abundance mapped to the genome or exon. Initial data representation was performed using MetaboAnalyst 6.0 software (www.metaboanalyst.ca). Then, the data were represented using a heatmap that allowed differentiation between non-treated and treated cells.

For identifying differentially expressed genes (DEGs) associated with CLO treatment and data visualization, iDEP 2.01 platform was used (Ge et al. 2018) using the DESeq2 package. Gene ontology (GO) enrichment analysis, including the Kyoto Encyclopedia of Genes and Genomes (KEGG) pathway (Kanehisa et al. 2021) were used to identify significant differences between phenotypes based on predefined gene sets, with a focus on biological pathways. Enrichment p-values were calculated based on one-sided hypergeometric test, which is then adjusted for multiple testing using the Benjamini–Hochberg procedure and converted to FDR.

### Western blotting

Protein equivalents from each sample were resolved in sodium dodecyl sulfate–polyacrylamide electrophoresis and were electrotransferred to PVDF membranes (Immobilon, Millipore) using a Bio-Rad Trans-Blot system according to the manufacturer’s instructions. For the study of protein expression by Western blotting, 3D HepaRG treated with CLO (1 or 5 mM) in the absence or presence of BA were used. Membranes were probed with adequate primary antibodies (Supplementary Table S2). Membranes were washed and incubated with the appropriate peroxidase-conjugated secondary antibodies and visualized as previously described (Donato et al. 2020). Band intensity was measured using ImageJ by selecting each band and subtracting background intensity. The corrected intensity was normalized to the corresponding total protein. Relative band intensity was calculated as the ratio of phosphorylated to total protein, ensuring consistent sample comparison.

### Statistical analysis

The results are provided as the mean ± standard deviation (SD). Experiments were performed by a minimum of three biological triplicates, each one including experimental replicates. The student’s t-test was run to make a comparison between two groups. For multiple comparisons, the statistical significance of the mean differences was evaluated by One-way ANOVA and Tukey’s multiple comparisons post-test or Dunnet’s test. *P* < 0.05 was assumed statistically significant and calculated using GraphPad Prism v8 (San Diego, CA, USA).

## Results

### HepaRG cells cultured in 3D collagen hydrogels with reduced DMSO concentration show increased expression of key hepatic genes compared to 2D culture

**Fig. 1:**
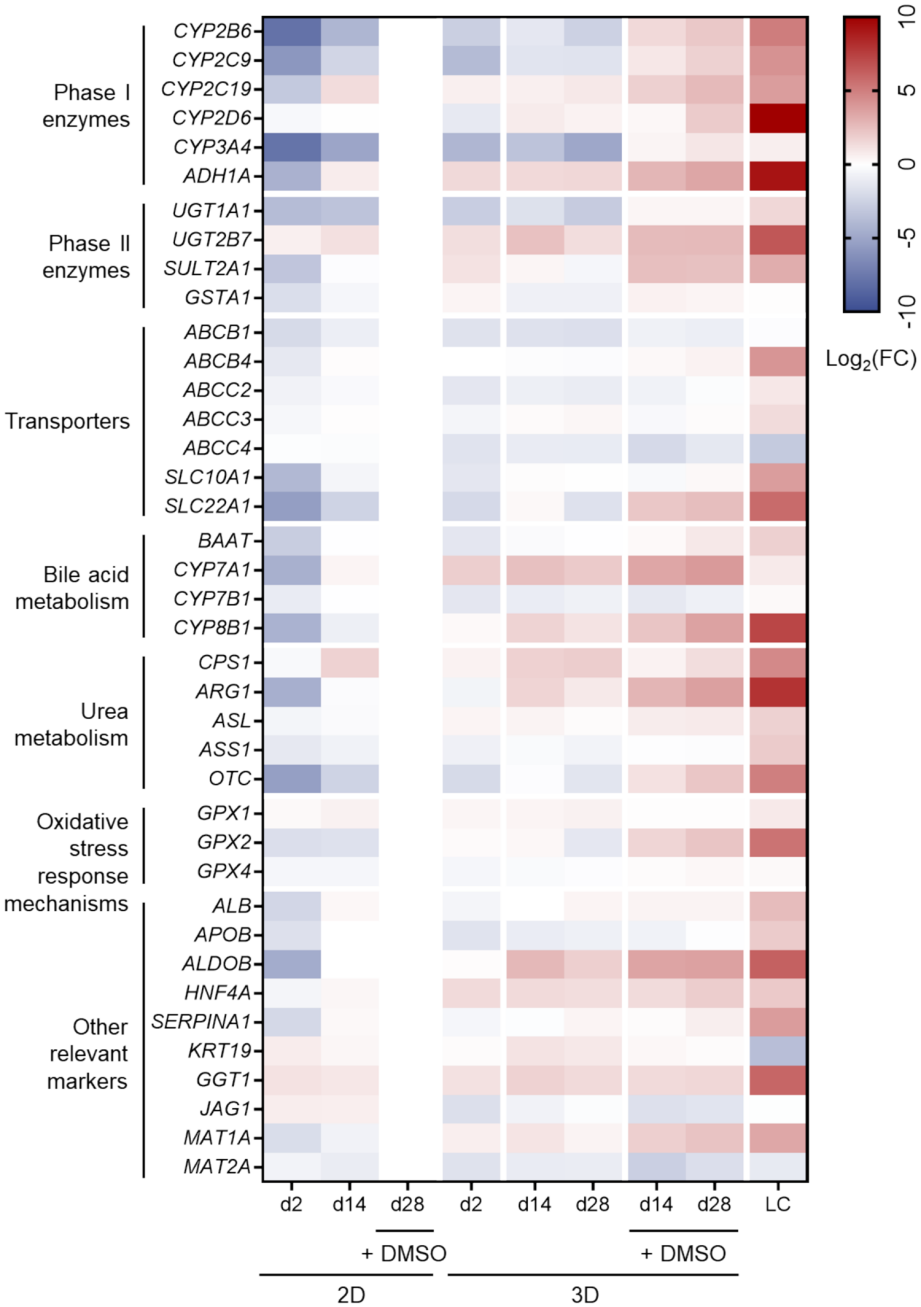
Expression of hepatic genes in HepaRG cells under different culture conditions. The heatmap shows the values of mRNA levels measured by RT-PCR for each time (2, 14, and 28 days) and culture condition (2D or 3D with absence or presence of DMSO) on a Log2 scale. The gene expression values for the standard condition (2D after 28 days of culture with 1.7% DMSO) were selected as the relative control. FC: relative change; LC: liver control

Expression profiles of 39 genes implicated in different liver processes, such phase I and II metabolism, transport function, BA and urea synthesis or antioxidant activities, exhibited by HepaRG cells cultured in 2D and 3D models are shown in (Fig. 1).

**Fig. 2:**
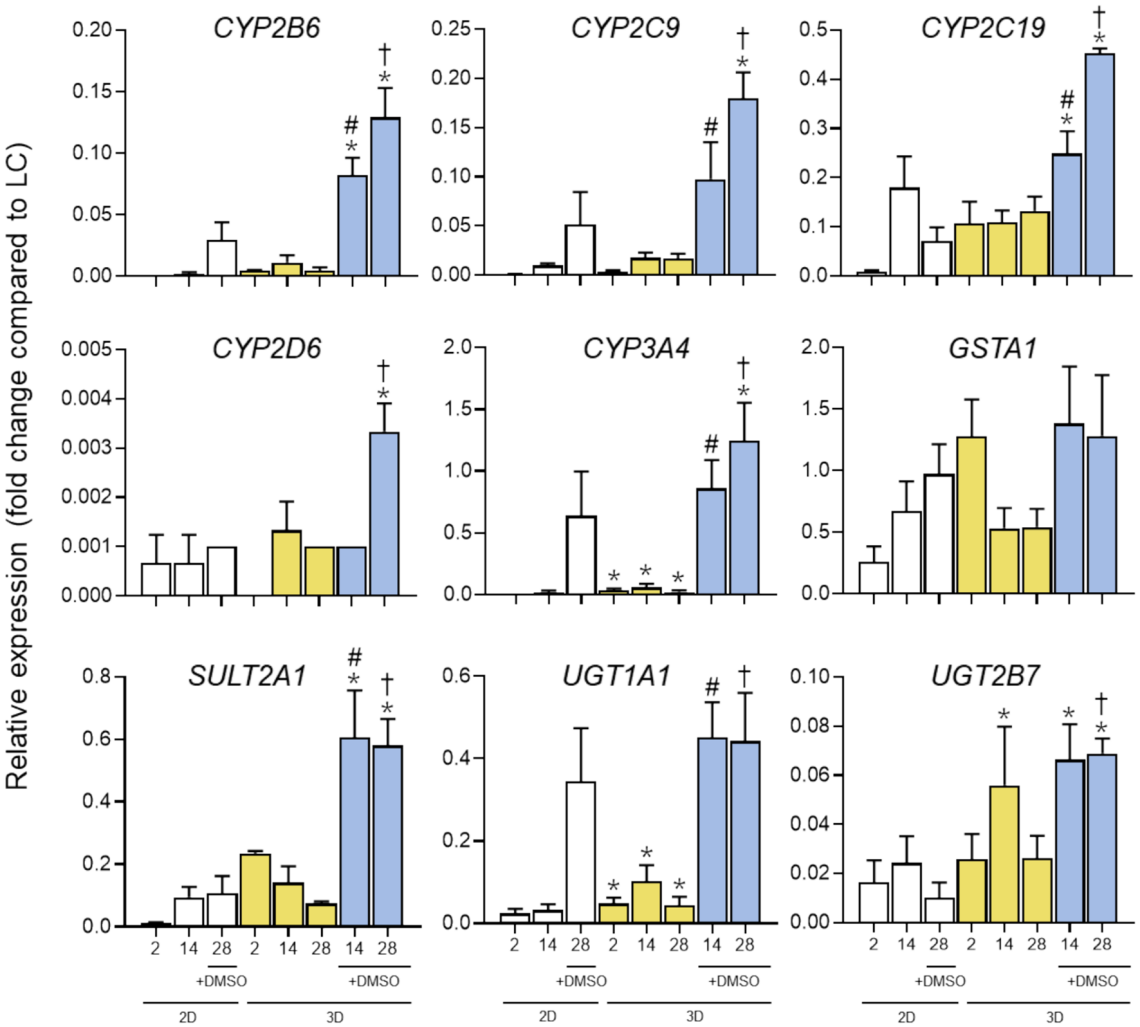
Gene expression of phase I and II enzymes in HepaRG cells under different culture conditions. mRNA levels were measured at day 2, 14 and 28 of culture. Data are expressed as relative change (FC) compared to human liver values (LC). ^*^At least *p* < 0.05 (compared to 2D + 1.7% DMSO at day 28); ^#^*p* < 0.05 (compared to 3D without DMSO Day 14); †*p* < 0.05 (compared to 3D without DMSO Day 28); (n = 3; ANOVA followed by Tukey’s multiple comparison test)

When cultured in 3D collagen hydrogels without DMSO, HepaRG cells upregulated a limited number of genes compared to the conventional 2D + 1.7% DMSO model.; however, when supplemented with 1% DMSO, most of the explored genes were upregulated in the 3D model, some of them even reaching the expression levels of a native liver. In particular, it is worth mentioning that mRNA levels of genes related to phase I (*CYP2B6, CYP2C9, CYP2C19, CYP3A4*) and phase II (*SULT2A1, UGT1A1, UGT2B7*) drug metabolism were significantly higher in HepaRG cells in 3D culture with 1% DMSO than in the 2D and DMSO-free 3D models (Fig. 2), highlighting the importance of the 3D collagen culture in the enhanced expression of these key hepatic genes. It was observed that high expression levels in the HepaRG cells in the 3D model were maintained up to 28 days of culture.

**Fig. 3:**
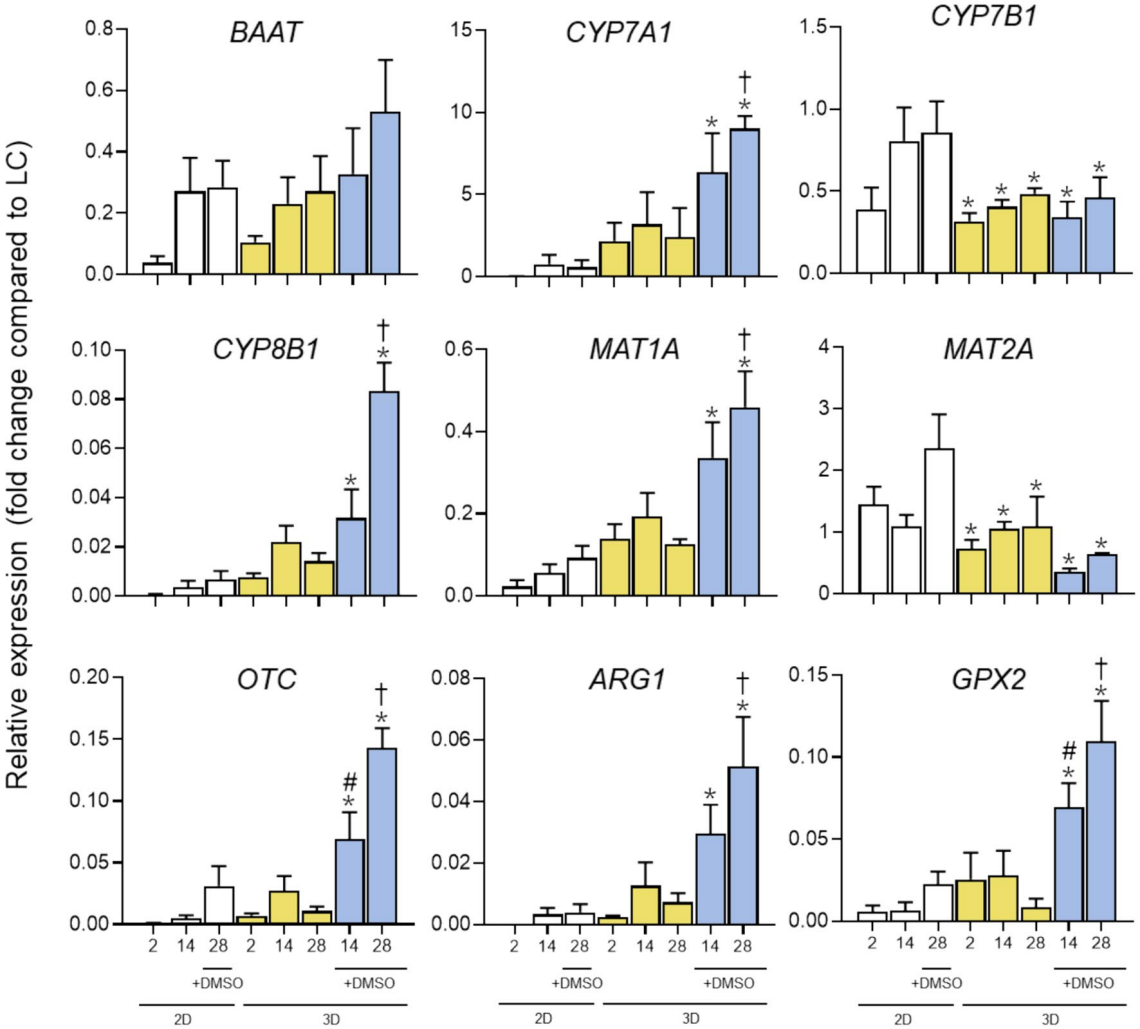
Gene expression of relevant hepatic genes in HepaRG cells under different culture conditions. Data are expressed as relative change (FC) compared to human liver values (LC). ^*^At least *p* < 0.05 (compared to 2D + 1.7% DMSO at day 28); ^#^*p* < 0.05 (compared to 3D without DMSO Day 14); †*p* < 0.05 (compared to 3D without DMSO Day 28); (n = 3; ANOVA followed by Tukey’s multiple comparison test)

In a similar way, BA synthesis genes *BAAT, CYP7A1* and *CY8B1* were also upregulated in the 3D + 1% DMSO condition compared to the conventional 2D model, except for *CYP7B1,* which was downregulated (Fig. 3). Other genes involved in typical liver functions such as ureagenesis (*OTC* and *ARG1*) or response to oxidative stress (*GPX2*) also showed higher expression in the 3D model with DMSO. Concerning *MAT1A* and *MAT2A*, which are respectively upregulated and downregulated in the adult liver, reproduced this expression pattern in a more appropriate way in the 3D + 1% DMSO condition than in the 2D culture. Similarly, other genes which were downregulated during the maturation of the liver, like *KRT19* or *JAG1*, also showed lower expression in the 3D model with DMSO compared to the 2D model, indicating a better hepatic differentiation process (Fig. 1).

### HepaRG cells cultured in 3D collagen show improved functionality compared to 2D

**Fig. 4:**
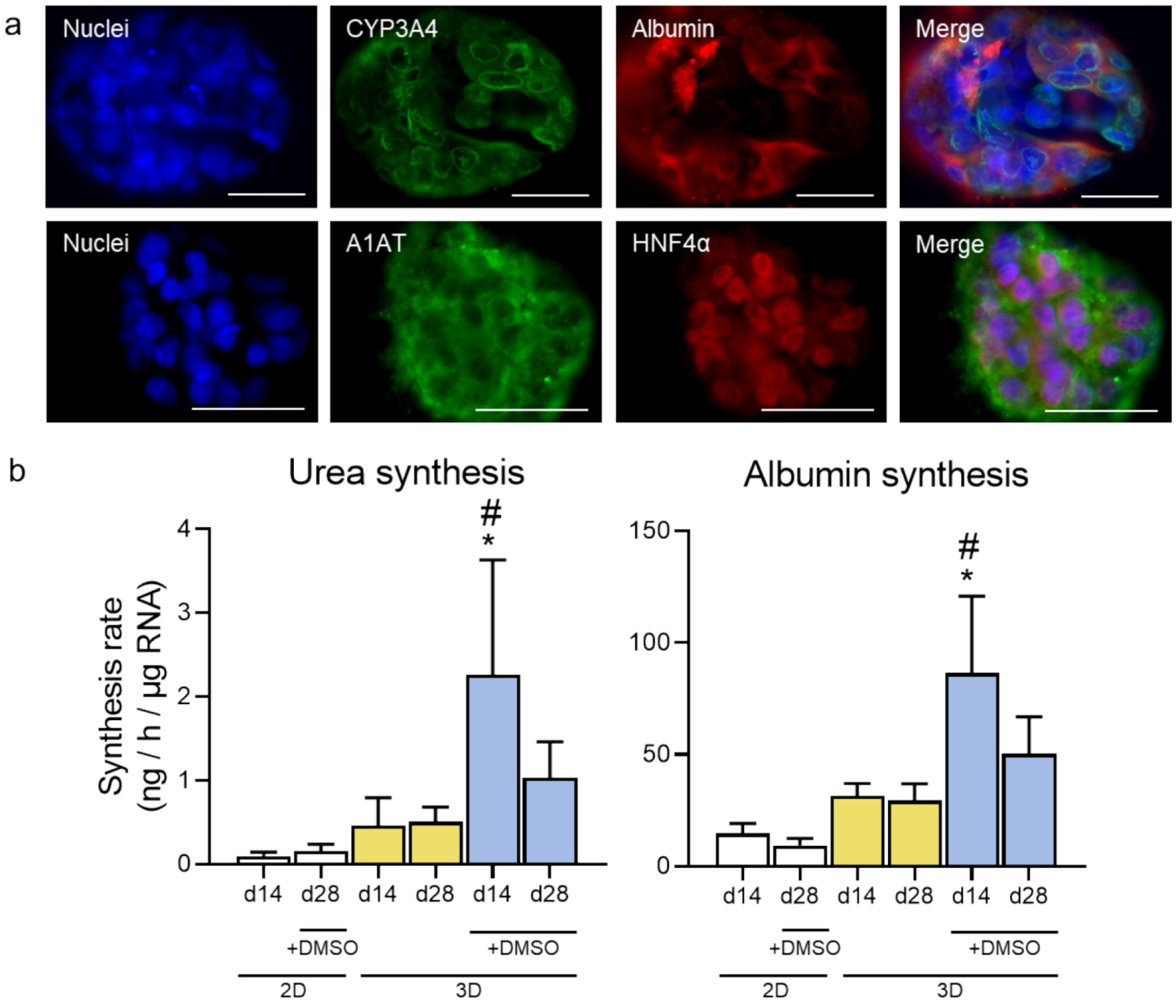
Characterization of HepaRG cells cultured in 3D collagen hydrogels. **A** Representative immunofluorescence images of the CYP3A4 (green) and albumin (red), and the A1AT expression (green) and HNF4 (red) expression after 14 days of culture in collagen hydrogels with 1% DMSO. Nuclei were identified by Hoechst 33,342 staining (blue). Scale bar (100 µm) applies to all the images. **B** Urea and albumin production in HepaRG cells cultured under different conditions. ^*^At least *p* < 0.05 (compared to 2D + 1.7% DMSO at day 28); ^#^*p* < 0.05 (compared to 3D without DMSO Day 14); (n = 3; ANOVA followed by Tukey’s multiple comparison test)

HepaRG cells cultured in 3D hydrogels and 1% DMSO expressed key liver markers such as hepatic nuclear factor 4 alpha (HNF4A), alpha 1 anti-trypsin (A1AT), albumin and CYP3A4 (Fig. 4a). Moreover, they also expressed other relevant hepatic proteins such as ApoB or UGT1A1 (Supplementary Figure S2).

To fully characterize the 3D model, different typical liver functions were examined. HepaRG cells cultured in 3D collagen hydrogels with 1% of DMSO showed higher production of urea and albumin than the 2D or the DMSO-free 3D conditions (Fig. 4b**)**. The highest rates of albumin and urea synthesis in the 3D model were observed on day 14 of culture and a subsequent decline were observed by day 28.

**Fig. 5:**
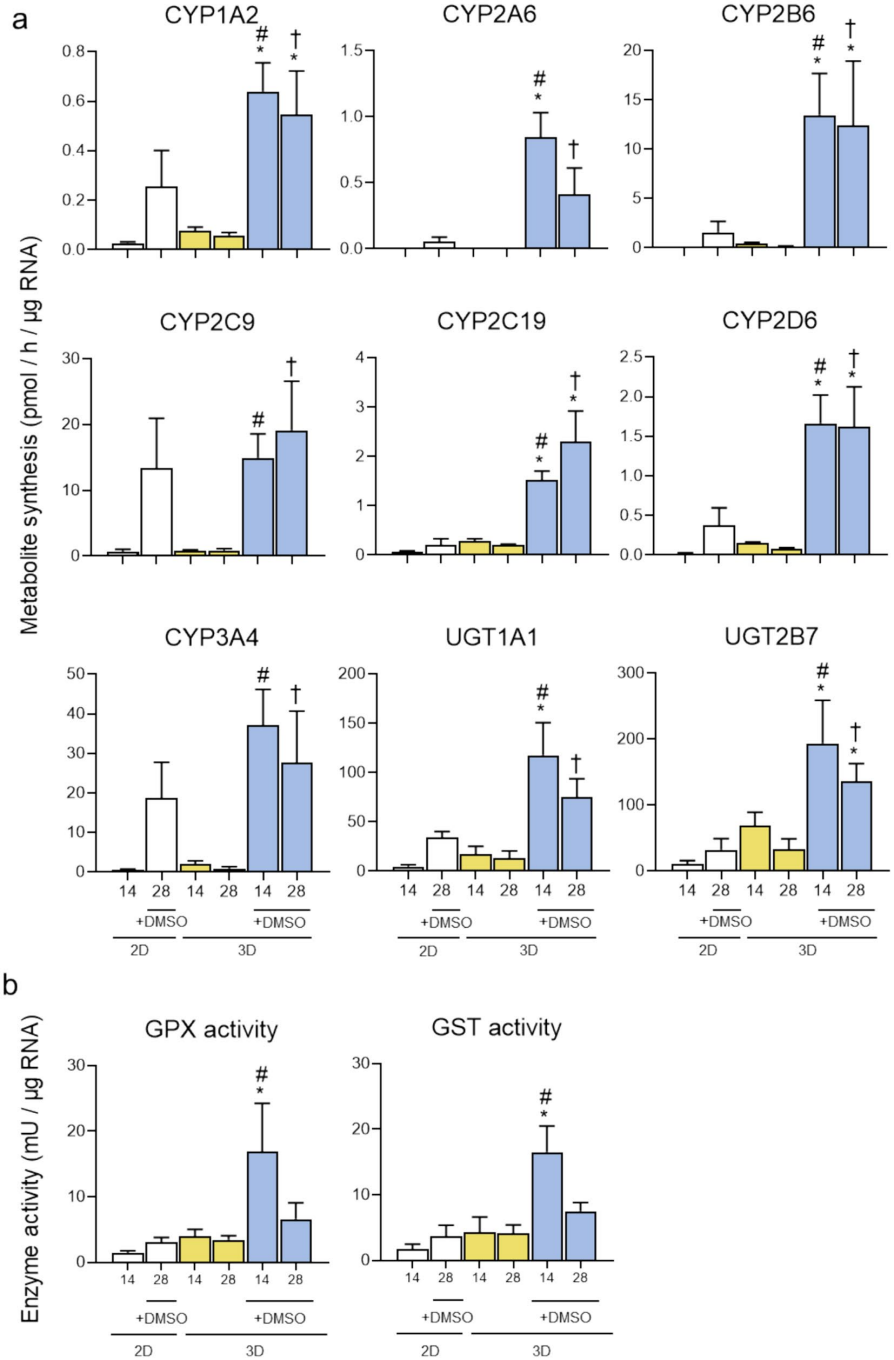
Enzyme activities of HepaRG cells cultured under different conditions. HepaRG cells were cultured for 14 or 28 days in 2D or 3D in the presence or absence of DMSO. **A** Activity levels of several CYP and UGT enzymes expressed as pmol of the corresponding metabolite formed per h and per µg of RNA. **B** Antioxidant enzymes activities expressed as mU per µg of RNA. ^*^At least p < 0.05 (compared to 2D + 1.7% DMSO at day 28); ^#^*p *< 0.05 (compared to 3D without DMSO Day 14); †*p* < 0.05 (compared to 3D without DMSO Day 28); (n = 3; ANOVA followed by Tukey’s multiple comparison test). CYP: cytochrome P450; DMSO: dimethyl sulfoxide; GPX: glutathione peroxidase; GST: glutathione S-transferase; UGT: UDP-glucuronosyltransferase

Phase I and phase II enzyme activities were also measured to characterize the detoxification capacity of the different models (Fig. 5a). HepaRG cells in the 3D model with DMSO showed measurable activity levels of different isoforms of CYPs, with CYP3A4, CYP2C9 and CYP2B6 showing the highest values. All CYP activities were higher than in the 2D or DMSO-free 3D models, being especially relevant the increases observed for CYP1A2, CYP2A6, CYP2B6, CYP2C19 and CYP2D6. Similarly, UGT1A1 and 2B7 activities were significantly higher in HepaRG under 3D cultures with 1% DMSO, although a slight reduction was observed after 28 days of culture (Fig. 5a). Glutathione peroxidase (GPX) and glutathione S-transferase (GST) activities were measured since they are relevant for the defense against oxidative stress (Fig. 5b). Again, HepaRG cells cultured in 3D 1% DMSO showed a significant increase in both activities compared to 2D culture or 3D cultures without DMSO. To fully characterize detoxification function, inducibility of CYPs was also evaluated (Supplementary Figure S3). After 48 h of exposure to three well-known inducers, phenobarbital, 3-methylcholanthrene and rifampicin, both gene expression and enzyme activities were significantly increased. Specifically, phenobarbital and rifampicin induced CYP2B6 and CYP3A4, while 3-methylcholanthrene induced CYP1A2.

**Fig. 6:**
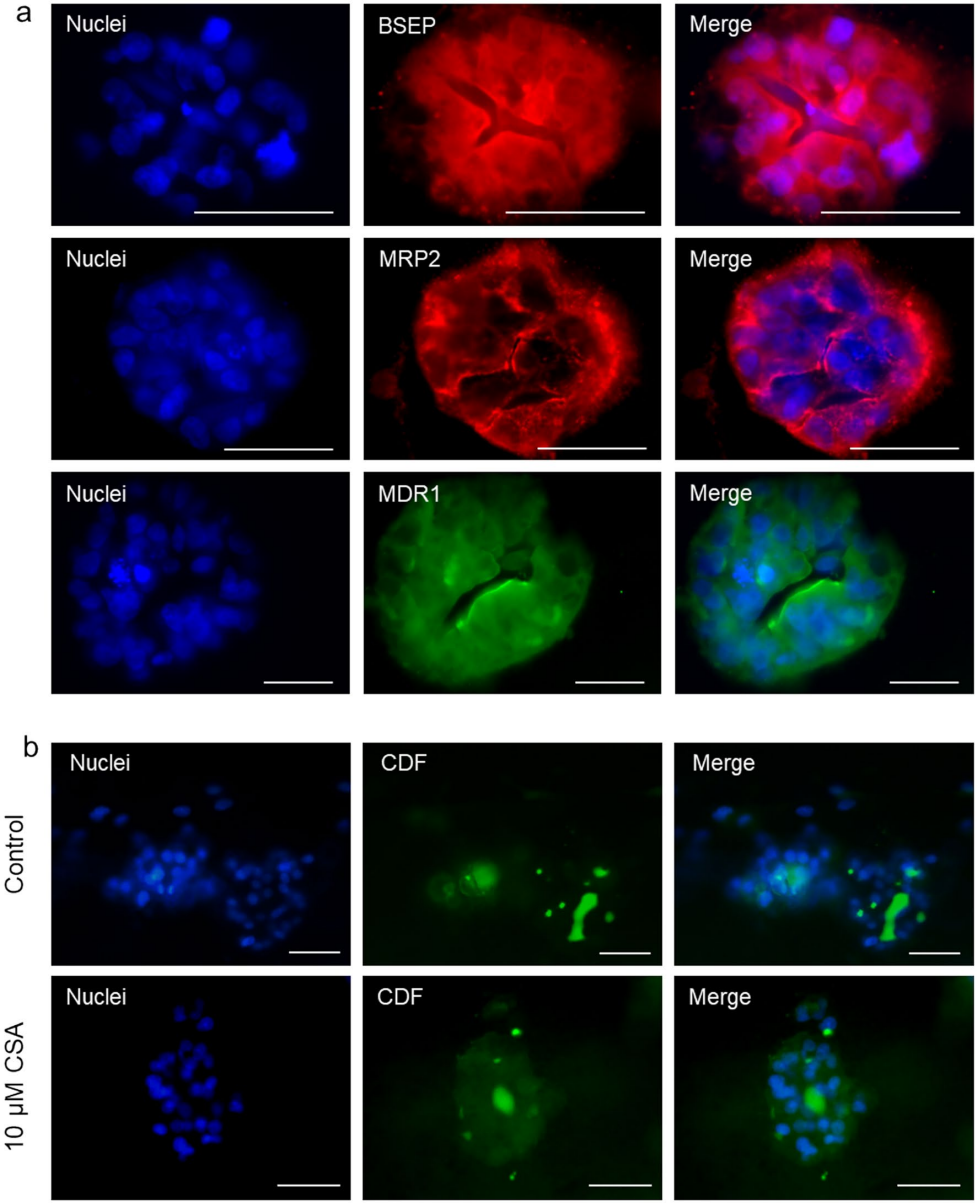
Transport function in HepaRG cells cultured in 3D collagen hydrogels. **A** Representative immunofluorescence images of the BSEP, MRP2 and MDR1 expression in 3D HepaRG cultures. Nuclei were identified by Hoechst 33,342 staining (Blue). **B** Representative fluorescent images showing the effects of cyclosporin A (CSA) on CDF efflux after 4 h of treatment**.** Nuclei were stained in blue (Hoechst 33,342). Scale bar (50 µm) applies to all the images

Transport function was also examined. HepaRG cells cultured in 3D expressed the BSEP, MRP2 and MDR1 transporters (Fig. 6a). The application of CDFDA assay revealed accumulation of green fluorescence into pseudo-canalicular structures formed inside the spheroids due to CDF clearance by MRP2. This finding was indicative of functional MRP2 activity in HepaRG cells that, as expected, was disrupted in the presence of cyclosporine A, an inhibitor of MRP2 transporter (Fig. 6b).

Based on their functional characterization, 3D HepaRG cells cultured in the presence of 1% DMSO for 14 days of culture were selected to further explore their suitability for hepatotoxicity studies.

### Suitability of 3D cultures of HepaRG for hepatotoxicity assessments

**Fig. 7:**
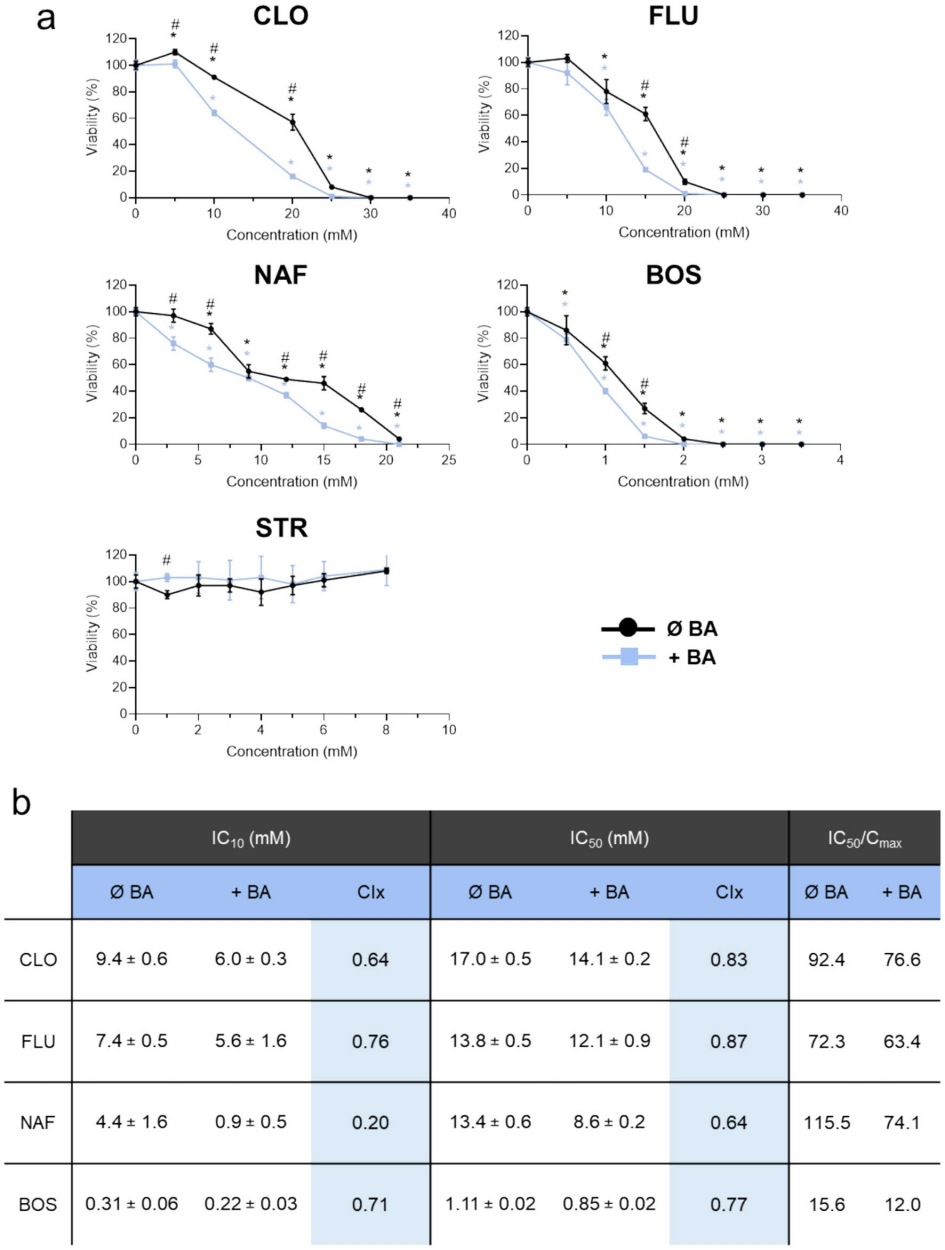
Cytotoxicity of test compounds in 3D HepaRG cells. **A** Intracellular ATP was determined in cells were exposed for 24 h to several concentrations of the drugs in absence or presence of bile acid (BA) mix. Viability is expressed as percentage of ATP content respect to the corresponding cells (with or without BA mix) no exposed to the drugs. ^*^At least *p *< 0.05 (compared to untreated cells in each condition (with or without BA, respectively, ANOVA followed by Dunnett’s test); ^#^*p* < 0.05 (compared each concentration in the absence or presence of BA; n = 3; Student’s t-test). **B** Analysis of the cholestatic potential of drugs. Concentrations that produce a decrease in viability of 10% (IC10) or 50% (IC50) in the presence or absence of BAs mixture are shown. Cholestatic index (CIx) is calculated as the ratio of IC10 or IC50 in the presence of BA respect the corresponding IC10 or IC50 value calculated in the absence of BA. BOS: bosentan; CLO: cloxacillin; FLU: flucloxacilln; NAF: nafcillin; STR: streptomycin

The utility of the 3D model to predict the hepatotoxicity associated to cholestatic antibiotics was examined. To this, the cells were exposed for 24 h to CLO, FLU and NAF, three β-lactam antibiotics with previous reports of cholestatic potential, as well as to BOS, a well-known cholestasis inducer, and STR, a non-hepatotoxic antibiotic, as positive and negative controls, respectively. To determine the cholestatic potential of these compounds, a cocktail of non-toxic concentrations of eight BAs was also included to generate a pro-cholestatic environment, and both intracellular ATP levels (Fig. 7) and release of LDH activity (Supplementary Figure S4) were measured as cytotoxicity parameters. For CLO, FLU, NAF and BOS, 3D HepaRG cells showed a concentration-dependent decrease in viability that was more accentuated in the presence of the BA cocktail, highlighting their cholestasis-associated hepatotoxicity. For STR, no effects in the cell viability were observed, even at the higher concentration tested (8 mM, which corresponds to > 200-fold of its therapeutical Cmax). Among the three β-lactam antibiotics, NAF showed the highest toxicity to HepaRG cells. The IC50 and IC10 values estimated for each compound in the absence or presence of the BA cocktail and the calculated CIx values (Fig. 7B) confirmed the cholestatic potential of CLO, FLU, NAF and BOS. Finally, long-term hepatotoxicity studies (up to 14 days of treatment) were also conducted to determine the suitability of the system to study chronic exposure to drugs (Supplementary Figure S5). As expected, an increase in compounds toxicity as a function of time was observed and, again, NAF proved to be more hepatotoxic than CLO and FLU.

### Gene expression in the 3D model after the exposure to cholestatic drugs

**Fig. 8:**
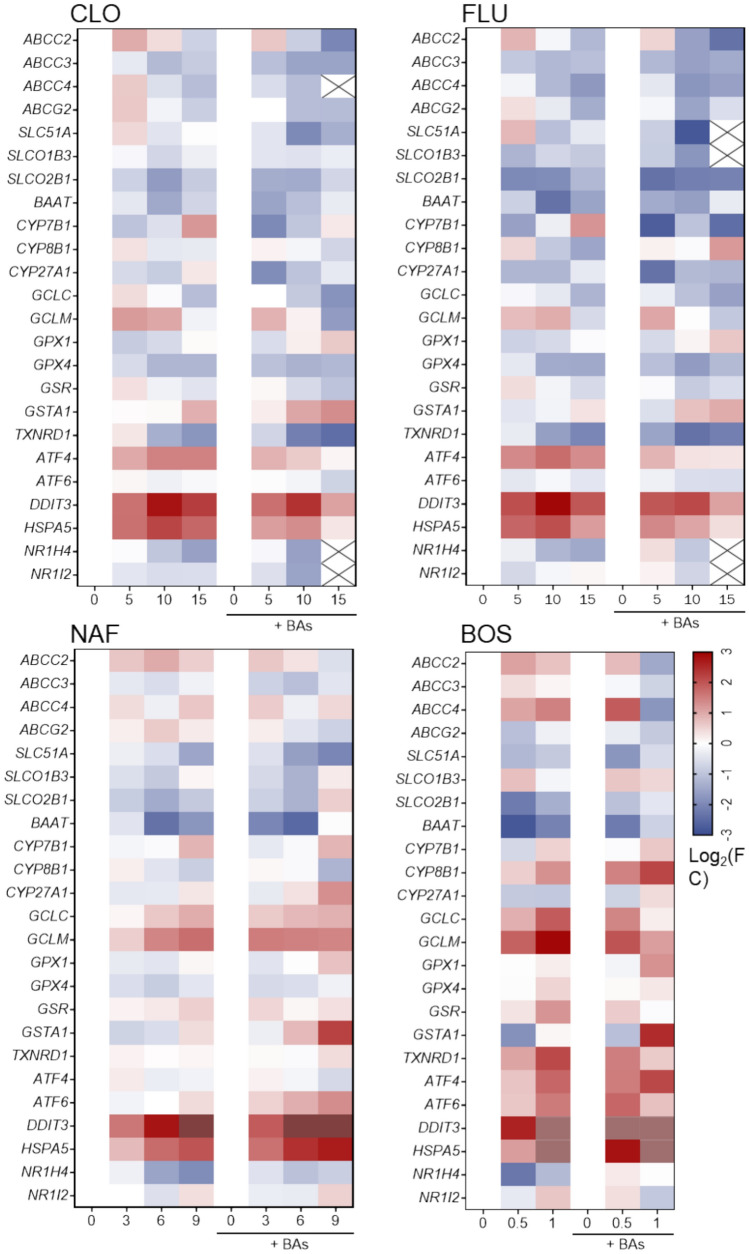
Transcriptomic changes in 3D HepaRG cells exposed to cholestatic drugs. Cells were exposed to cloxacillin (CLO 5, 10, 15 mM), flucloxacillin (FLU, 5, 10, 15 mM), nafcillin (NAF 3, 6, 9 mM) and bosentan (BOS 0.5, 1 mM) in the absence or presence of bile acids (BA) for 24 h and mRNA levels were measured by RT-PCR. The heatmap shows the values on a Log2 scale calculated respect to the corresponding untreated cells. Brown: upregulation out of scale; X: non-detected. BOS: bosentan; CLO: cloxacillin; FLU: flucloxacilln; NAF: nafcillin

To investigate the underlying mechanisms in the toxicity of these compounds, alterations in the expression levels of several families of genes potentially related to cholestatic liver damage, such as apical (*ABCC2, ABCC3, ABCC4, ABCG2*) and basolateral (*SLC51A, SLCO1B3, SLCO2B1*) transport, BA metabolism (*BAAT, CYP7B1, CYP8B1, CYP27A1, NR1H4, NR1I2*), and cellular response to oxidative stress (*GCLC, GCLM, GPX1, GPX4, GSR, GSTA1, TXNRD1*) and endoplasmic reticulum (ER) stress (*ATF4, ATF6, DDIT3, HSPA5*) were examined. Figure 8 summarizes transcriptome profiles of 3D HepaRG cells treated for 24 h to several concentrations of CLO, FLU, NAF and BOS, in the absence or presence of BAs. Similar transcriptomic changes were found for CLO and FLU, usually promoting a downregulation of all these genes, except for *CYP7B1*, *GSTA1*, *ATF4*, *DDIT3* and *HSPA5*, which were upregulated, at the higher concentrations of the compounds. Co-exposure of CLO and FLU with BAs moderately decreased the expression of most genes, exacerbating the downregulating effect. In contrast, after treatment with NAF and BOS, most of the explored genes were upregulated, at least at the higher concentrations of these drugs, and these effects were higher in the presence of BAs.

### Exploring the mechanisms of CLO-induced toxicity

In order to decipher the major pathways implicated in CLO-induced toxicity, a RNAseq analysis was performed in 3D HepaRG cells exposed to the drug in the presence or absence of BAs. The principal component analysis (PCA) scores plots showed an almost complete separation between treated and non-treated cells in the presence or absence of BAs (Supplementary Figure S6). At the lowest concentration of CLO tested (7.5 mM), a clear separation between cells with or without BAs was observed.

**Fig. 9:**
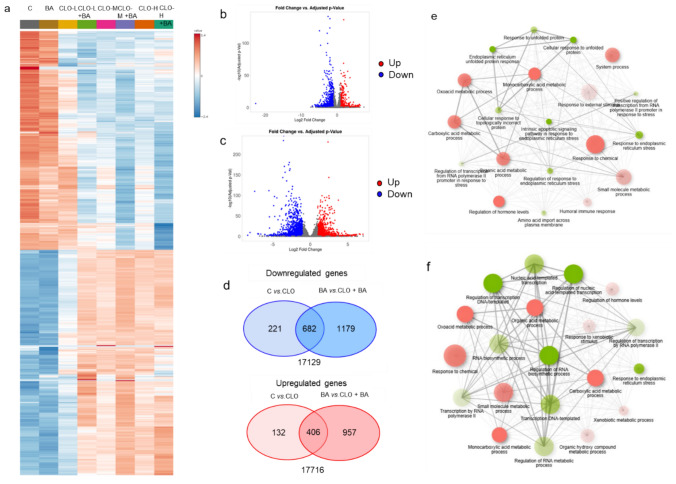
Transcriptional analysis of cloxacillin-treated 3D HepaRG cells assessed by RNAseq. **A** Heatmap depicting the sample-level expression of the top 1000 up-regulated and down-regulated differentially expressed genes (DEGs) in different experimental conditions (concentrations from low (L, 7.5 mM), medium (M, 10 mM) and high (H, 12.5 mM) of cloxacillin (CLO) in the absence or presence of bile acids (BA). RNA-seq volcano plots for the comparison between cells treated in culture medium (Control and CLO 7.5 mM) **b** and in medium with BA cocktail (BA and CLO7.5-BA) **c** in the 3D HepaRG model. Genes with an adjusted *p*-value < 0.05 and absolute log2 fold change > 2 were highlighted as differentially expressed genes. **D** Venn diagrams showing the number of up-regulated and down-regulated DEGs altered by cloxacillin in the 3D HepaRG model compared to their paired controls (control and BA). Networks of enrichment KEGG pathways of downregulated genes in cell treated with cloxacillin 7.5 mM compared to control cells **e** and cells treated with the same concentration of CLO in the presence of BA, compared to cells culture in the presence of the BA cocktail **f**

Heatmap of different conditions showed a clear separation between non-treated (control, BA) and CLO-treated cultures (Fig. 9a). To further understand the transcriptional changes induced by CLO, we focused on the lowest concentration used (7.5 mM) in the presence or absence of the BA cocktail. A total of 1441 (538 upregulated and 903 downregulated) genes were significantly changed after CLO treatment compared to non-treated cultures (Fig. 9b). In the presence of BA mix, more genes significantly changed: 1363 upregulated genes and 1861 downregulated genes (Fig. 9c). Venn diagrams showed that most of these altered genes were commonly altered in both conditions (Fig. 9d). To elucidate the distinct biological roles of regulated genes in 3D HepaRG cultures, we performed GO enrichment analysis on DEGs identified in CLO-treated cultures in the presence or absence of BA (Fig. 9e and f, respectively). In the absence of BA, CLO mainly produced a downregulation of monocarboxylic/oxoacid/carboxylic/organic acid metabolic processes and pathways related to responses to external stimulus such as chemicals. Response to ER stress was an upregulated pathway, which would indicate the activation of the unfolded protein response after CLO-treatment (Fig. 9e, Supplementary Figure S7a). In the presence of BA, similar biological pathways were altered although the number of genes and the fold-enriched values were significantly higher (Fig. 9f, Supplementary Figure S7b). Xenobiotic metabolic process and responses to xenobiotic stimulus and chemicals were also significantly changed, which would indicate alteration in CYPs and phase II conjugation enzymes due to damage to the detoxification systems. Finally, KEGG analysis of overlapped genes showed glycolysis/gluconeogenesis, bile secretion and drug metabolism pathways as the top enriched pathways common in the presence or absence of BA after CLO treatment (Supplementary Figure S8a). On the other hand, the analysis of overlapping reactome pathways revealed significant alterations in ADME (absorption, distribution, metabolism and excretion) processes, different signaling pathways and ER stress (Supplementary Figure S8b).

**Fig. 10:**
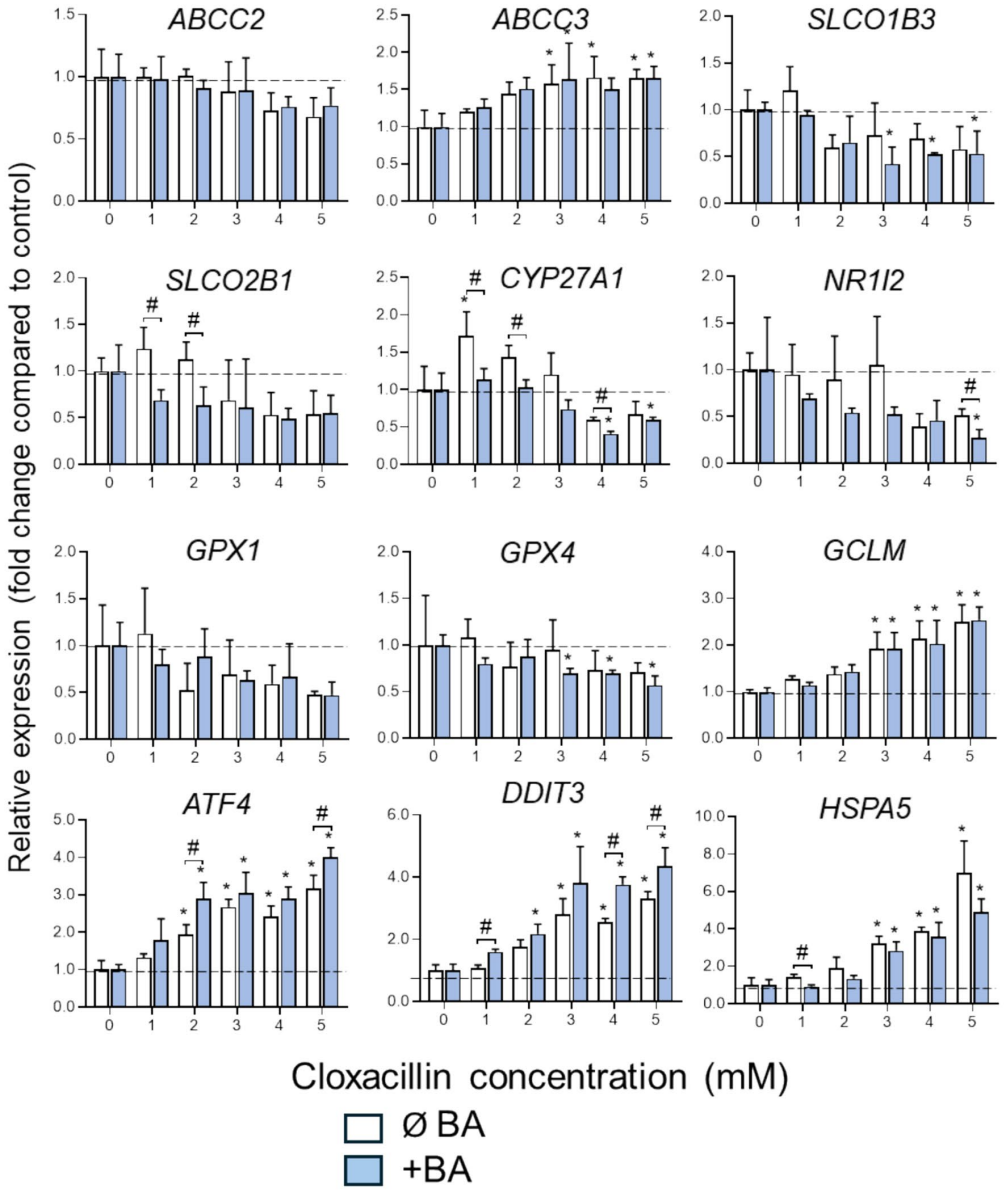
Gene expression of relevant cholestatic markers in HepaRG cells after cloxacillin exposure. 3D HepaRG cells were treated with different concentrations of cloxacillin (CLO) for 24 h in the absence or presence of bile acids (BA) and mRNA levels were measured by RT-PCR. Data are expressed as relative change (FC) compared to untreated cultures. ^*^At least *p* < 0.05 (compared to untreated cells in each condition (with or without BA, respectively, ANOVA followed by Dunnett); ^#^*p* < 0.05 (compared each concentration in the absence or presence of BA; n = 3; Student’s t-test

The effects of CLO at lower concentrations on the expression of genes implicated in different functional liver processes were also analyzed (Fig. 10). Regarding transporters, a significant decrease in the gene expression levels of *SLCO1B3* was observed, along with an increase in *ABCC3* expression. Genes involved in BA metabolism (*CYP27A1, NR1I2*) and oxidative stress response (*GPX4*) also showed a dose-dependent significant decrease in their expression, except for *GCLM* which was upregulated. Finally, the expression of ER stress response genes (*ATF4, DDIT3, HSPA5*) showed a concentration-dependent increase. Overall, for these concentrations of CLO, co-treatment with BAs did not significantly influence changes in gene expression, except for CYP27A1, whose expression was further repressed in the presence of BAs, and *ATF4* and *DDIT3*, whose expressions increased in their presence. Additionally, the effects on phase I enzymes were also assessed at both the gene expression and the enzyme activity levels (Supplementary Figure S9). For CYP2B6 and CYP3A4 concentration-dependent induction were observed up to 5–7.5 mM CLO, whereas no significant effects were found on CYP2C9 and CYP2C19.

**Fig. 11:**
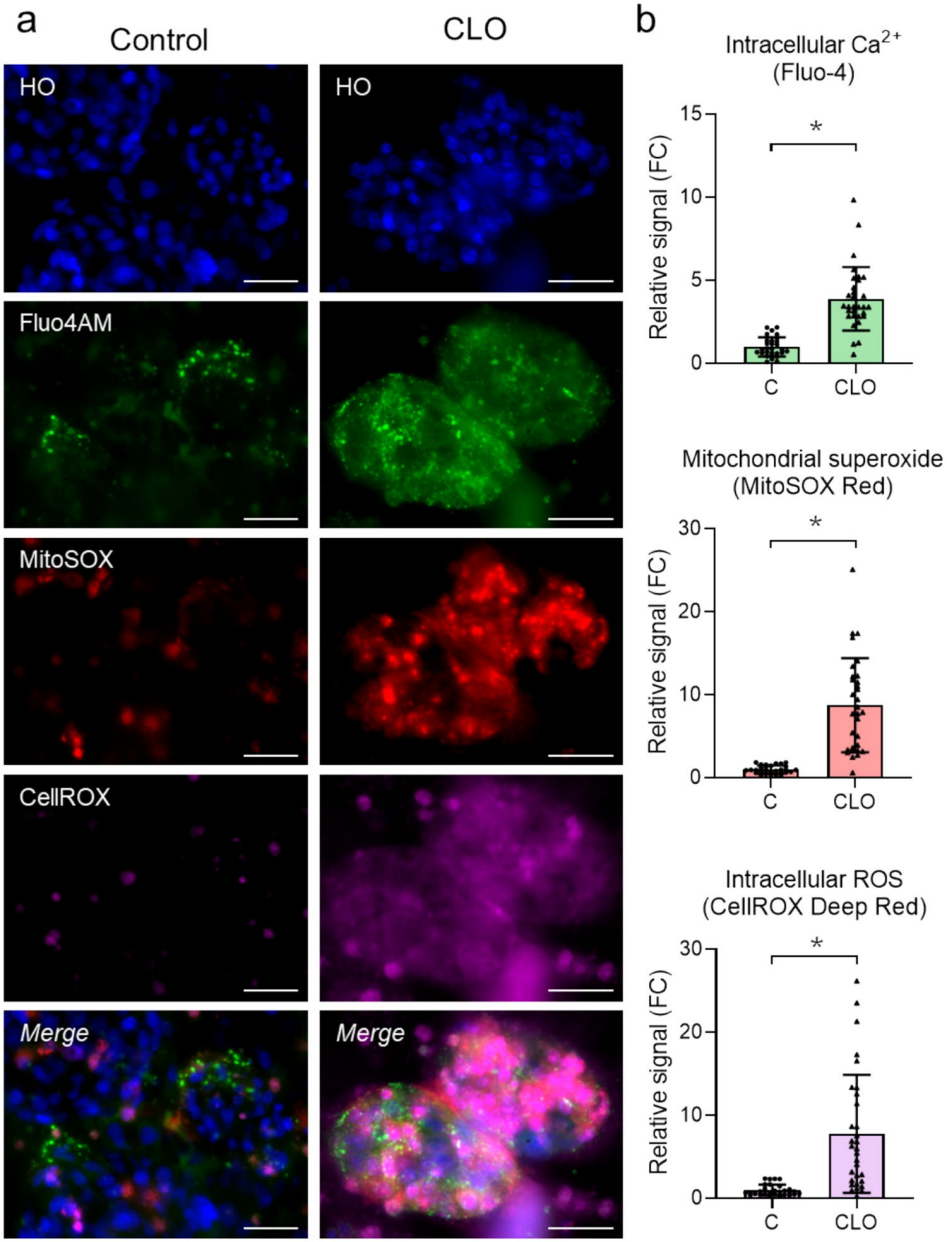
Mechanistic assessment of cloxacillin-induced hepatotoxicity by high-content screening. 3D HepaRG cells were exposed to 12.5 mM of cloxacillin (CLO) for 24 h and the effects on ROS production, intracellular calcium concentration and mitochondrial superoxide production were analyzed. **A** Representative images of increased intracellular calcium concentration (Fluo4AM in green), mitochondrial superoxide (MitoSOX in red) and ROS (CellROX staining in pink) after treatment with CLO. **B** Quantification of Fluo4AM, MitoSOX and CellROX fluorescence in untreated cells (control) and cells treated with CLO and. *At least *p* < 0.05 (n = 3; parametric Student’s t-test)

Several indicators of cell toxicity were also examined by high-content screening. CLO induced an increase in intracellular calcium concentration, mitochondrial superoxide and ROS, which suggested the role of oxidative stress in CLO-induced toxicity (Fig. 11). Accordingly, CLO produced concentration-dependent changes in the gene expression of several antioxidant enzymes (Supplementary Figure S10).

### Signaling pathways modulated by CLO

**Fig. 12:**
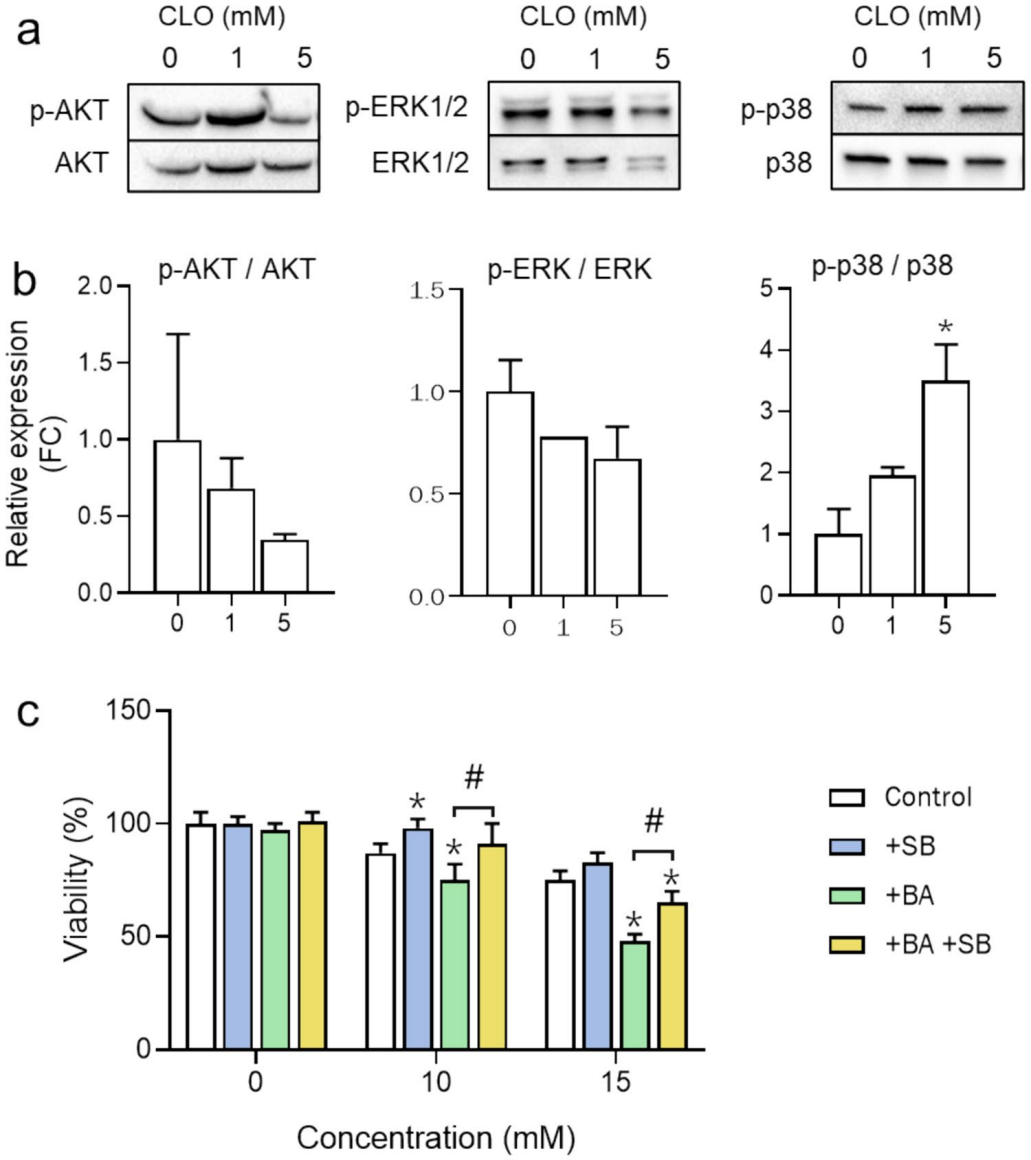
Role of p38 pathway in cloxacillin-induced toxicity in 3D HepaRG cells. Representative western blots **A** and quantification **B** of P-AKT/AKT, P-ERK1/2/ERK1-2 and P-p38/p38 after 24 h-treatment with different concentrations of cloxacillin (CLO) in 3D HepaRG cells. **C** Viability, assessed by ATP assay, after 24 h incubation with CLO in the absence or presence of BAs and/or the selective inhibitor of p38 adezmapimod (SB 10 µM). ^*^At least *p* < 0.05 (compared to control cells); ^#^*p* < 0.05 (compared to cells treated in the presence of BA (+ BA)); (n = 3; ANOVA followed by Tukey’s multiple comparison test)

To study the signaling pathways underlying CLO toxicity, a protein analysis was performed using Western blot. After 24 h of treatment with low, non-cytotoxic, concentrations of CLO, a significant decrease in the phosphorylation levels of AKT and ERK was observed (Fig. 12A and B). Both proteins are related to cell survival and proliferation, and this decrease was even more pronounced in the presence of BAs. On the other hand, p38 showed an increase in its phosphorylation, which was also accentuated in the presence of BAs (Fig. 12A and B). To determine the specific role of p38 in CLO-induced toxicity, adezmapimod (SB203580), a specific competitive inhibitor of p-p38, was used concomitantly with 24 h CLO treatment, in the absence and presence of BAs. Co-treatment with adezmapimod resulted in an increase in cell viability compared to cells treated with CLO alone, both in the absence or presence of BAs, which would confirm the role of this kinase in the induction of cytotoxicity (Fig. 12C).

## Discussion

In the present study we prepared 3D cultures of HepaRG cells encapsulated in collagen type I hydrogels with the aim of promoting cell functionality and reducing the culture time and concentration of DMSO required for their differentiation. Collagen type I is very abundant in the liver extracellular matrix and plays an important role in cell growth, differentiation, and survival and tissue organization (Bedossa and Paradis 2003). HepaRG cells embedded in 3D collagen-based scaffolds exhibited typical hepatocyte markers, as revealed by immunofluorescence examination of albumin, A1AT or MRP2. An analysis of gene expression profile showed that HepaRG in 3D culture express hepatic genes involved in key liver functions such as phase I and phase II metabolism of drugs, transport of xenobiotics and endogenous molecules, synthesis of plasma proteins, lipid metabolism, ammonia detoxification or antioxidant defense. In general, expression levels of typical hepatic genes were higher than those found in HepaRG differentiated in conventional 2D cultures. In agreement with this, 3D cultures showed better functional capacity as reflected by higher urea and albumin synthesis rates and higher activity levels of CYPs, conjugation enzymes and antioxidant enzymes.

A major feature of the differentiation protocol of HepaRG cells in 2D cultures is the presence of DMSO in the medium. DMSO is essential for both maturation and maintenance of differentiated phenotype of HepaRG in 2D cultures and its withdrawal leads to a rapid and drastic decay of typical hepatic functions (Aninat et al. 2006; Kanebratt and Andersson 2008). It has been reported that high DMSO concentrations, as required for HepaRG differentiation under 2D conditions, induce some CYP activities (e.g. CYP3A4, CYP2C9 or CYP2B6) but repress various typical hepatic functions (e.g. albumin synthesis and urea detoxication) (Aleksandrova et al. 2016; Aninat et al. 2006; Hoekstra et al. 2011). Other authors have previously explored different DMSO-free 3D culture systems for HepaRG differentiation. Most of them consist of self-assembled aggregates of cells which form spheroids that are subsequently encapsulated or not in a biomaterial such as alginate or collagen and maintained under stirred or static conditions (Coulet et al. 2024; Gunness et al. 2013; Leite et al. 2012; Liao et al. 2022; Mueller et al. 2014; Rebelo et al. 2015). Alternatively, as in our proposal, HepaRG cell suspension can be embedded directly into a hydrogel to create an extracellular environment suitable for promoting cell–cell and cell–matrix interactions and allowing diffusion of nutrients and soluble factors through the hydrogel (Chowdhury et al. 2024; Rose et al. 2022). There is a consensus that 3D cultures favor cell differentiation and maintenance of biosynthetic functions (Gunness et al. 2013; Rebelo et al. 2015; Rose et al. 2022).

To our knowledge, this is the first study that comprehensively analyzed the role of DMSO in gene expression profiles and functionality of HepaRG cells in a 3D culture system. We found that HepaRG cells embedded in collagen and maintained in the presence of 1% DMSO reached a better hepatocyte-like phenotype than 3D HepaRG cultured in DMSO-free medium or conventional 2D cultures with 1.7% DMSO, as evidenced both at transcriptional and functional levels. In their 3D HepaRG model based on DMSO-free collagen hydrogels, Rose et al. (2022) observed discrete increases of around twofold in the gene expression of some CYPs, such as CYP1A2, CYP2D6 or CYP2C9, compared to the conventional 2D model; however, this transcriptional improvement was not found at the functional level, with very similar activity levels between both models. In contrast, our 3D model showed 2- to tenfold increases over the 2D model in gene expression and activity of all the CYP enzymes. Moreover, these cells showed a generalized increase, compared to the 2D model, in the expression and/or activity of various UGT, SULT, and GST enzymes. Although previous studies had already indicated that maintaining HepaRG cells in 3D culture favored the gene expression of conjugation enzymes (Rose et al. 2022), the improvement of their activity levels had not been observed. Thus, DMSO seemed a key factor contributing to promote differentiation of HepaRG not only in 2D but also in 3D cultures. The underlying mechanisms of these effects need further investigation, but they have been related to both transcriptional and epigenetics modifications induced by DMSO during HepaRG differentiation (Dubois-Pot-Schneider et al. 2022).

Given their improved properties and metabolic capacity, we explored the potential utility of this 3D HepaRG model for DILI studies. To this end, we examined the effects induced by three β-lactam antibiotics (CLO, FLU, NAF) with previous reports of cholestatic liver injury in patients (Alam et al. 2012; Angheleanu and Swart 2021; Faragalla et al. 2022; Guido et al. 2017; Wing et al. 2017). Cytotoxicity induced by each drug alone or in combination with a mixture of non-toxic concentrations of BAs was comparatively analyzed. This strategy allows to detect BA-selective sensitization towards toxic effect of cholestatic drugs and is commonly used in vitro to distinguish cholestatic hepatotoxicity from non-cholestatic hepatotoxicity (Chatterjee et al. 2014; Gijbels et al. 2020; Rose et al. 2022). Consistent with their cholestatic potential, CLO, FLU and NAF induced higher cytotoxicity in the presence of BA and showed a Clx < 0.8. This synergistic toxicity, not previously reported for these antibiotics, may be associated to the ability of cholestatic compounds to impair BA transport that leads to potential alterations of BA homeostasis resulting in intracellular accumulation of toxic BA concentrations (Chatterjee et al. 2014; Rose et al. 2022). A similar effect was observed with BOS, a model cholestasis inducer, but not with STR, a non-hepatotoxic antibiotic, suggesting the potential utility of our 3D model to discriminate cholestatic compounds. Of note, in our model cholestatic effects (Clx < 0. 8) were detected after 24 h of exposure to the drugs, whereas other previous proposals based in 3D HepaRG models used longer treatments (up to 14 days) to screen for the cholestatic liability of drugs (Drees et al. 2025; Hendriks et al. 2016; Rose et al. 2022).

The potential mechanisms involved in cholestatic effects of β-lactam antibiotics remain unclear. Some in vitro studies using isolated bile canalicular membrane vesicles or hepatocytes in sandwich configurations revealed the interference of these drugs on taurocholate uptake and efflux (Barber et al. 2015; Horikawa et al. 2003; Wolf et al. 2010). Other authors suggested the role of ER stress induction and activation of HSP27 and PI3K/AKT pathways in bile canaliculi dilatation and cholestatic damage induced by CLO, FLU and NAF in primary hepatocytes or HepaRG cells in 2D cultures (Burban et al. 2018, 2017). In agreement with these findings, we observed a marked upregulated expression of genes related to ER stress response, such as *ATF4*, *DDIT3* and *HSPA5*. Moreover, marked alterations were also found in the expression profile of genes related to oxidative stress and CYP enzymes. Comparative analysis of the effects of CLO, FLU and NAF on gene expression in our HepaRG 3D model, revealed that CLO and FLU modified the gene expression landscape in a very similar way, suggesting that hepatotoxic effects induced by both drugs may be mechanistically similar.

RNA-seq analysis of liver cells exposed to CLO revealed a transcriptional signature characteristic of cellular stress, notably the activation of the ER stress response. GO enrichment showed significant upregulation of pathways associated with the unfolded protein response, including response to ER stress, regulation of transcription by RNA polymerase II, and amino acid import that have been previously described for cholestatic drugs (Stieger et al. 2000; Szczesna-Skorupa et al. 2004; Wen et al. 2022). These changes suggest a compensatory mechanism aimed at restoring proteostasis and metabolic adaptation. Concomitantly, there was a broad downregulation of pathways involved in core hepatic metabolic functions, including monocarboxylic acid metabolism, xenobiotic metabolic processes, and regulation of hormone levels. This pattern reflects a shift away from normal liver-specific roles, such as detoxification and endocrine regulation, toward damage containment and survival. The suppression of xenobiotic and small molecule metabolism further indicates potential impairment of the liver’s detoxification capacity, which may exacerbate cellular injury. In fact, the reduction of xenobiotic metabolism has been described in human samples of obstructive cholestasis (Chai et al. 2015a, 2015b) and could further indicate a possible impairment of the liver’s detoxification capacity, which in turn could exacerbate cellular injury. Taken together, these results highlight a coordinated stress response in hepatocytes, characterized by stress-mediated transcriptional reprogramming of the ER and metabolic alterations, commonly observed in DILI (Zhang et al. 2022). The presence of BAs during incubation with CLO significantly amplified transcriptional responses in HepaRG cells. BAs are endogenous ligands of several nuclear receptors, including FXR and PXR, which regulate genes involved in xenobiotic metabolism, BA homeostasis, and inflammation (Chiang 2013; Zollner et al. 2006). When a drug is administered in the context of elevated BA levels, as occurs in cholestasis, these receptors can be synergistically activated, leading to broader and more robust transcriptional changes than would occur with the drug alone (Wagner et al. 2009). In addition, it has been described how BAs induce cellular stress pathways, such as increased ROS and ER stress, which further activate transcription factors, such as NRF2 or nuclear factor κB (Chiang 2013). Furthermore, the reactome analysis confirmed the modulation of ADME processes which allows enhancing mechanistic understanding of compound accumulation, metabolic activation, or detoxification in drug-induced toxicity.

Finally, BAs can activate different signaling pathways, such as those involving the AKT, ERK, p38, and JNK proteins (Yu et al. 2018), and it has been described how different cholestatic drugs can also activate these signaling pathways (Sharanek et al. 2014). Treatment with CLO significantly reduced the phosphorylation levels, and therefore the activity, of AKT and ERK, kinases involved in cell survival and proliferation, with this effect being even more pronounced in the presence of exogenous BAs. Concomitantly, this treatment also induced the phosphorylation of p38, which is involved in cell apoptosis. These findings are in accordance with previous studies which showed that other antibiotics such as FLU induce a cholestatic phenotype in human hepatocytes through the activation of heat shock protein 27, associated with the p38 and PI3K/AKT signaling pathways (Burban et al. 2017). Similarly, cyclosporine has been shown to generate oxidative stress by triggering the activation of the PKC/ERK/JNK/p38 signaling pathway and the subsequent internalization of MRP2 and BSEP (Sharanek et al. 2014). Inhibition of the P-p38 signaling pathway reduced CLO-induced cytotoxicity which would confirm, to our knowledge, for the first time, the role of this pathway in CLO-induced cholestasis.

In conclusion, in this work we have developed and characterized a competent 3D model of HepaRG cultured in type I collagen hydrogels that has allowed us to reproduce the cholestatic toxicity of three known cholestatic antibiotics. This 3D hepatic model shows great promise in enabling comprehensive mechanistic investigations into cholestatic DILI associated to these drugs. In the future, the application of this HepaRG model could allow earlier identification of drug candidates which cause DILI, thus reducing attrition earlier in the drug development process.

## Supplementary Information

Below is the link to the electronic supplementary material.

Supplementary file1 (DOCX 1613 KB)

## Data Availability

Data will be accesible under request.

## Abbreviations

2D: Two-dimensional
3D: Three-dimensional
ADME: Absorption, distribution, metabolism and excretion
BAs: Bile acids
BOS: Bosentan
BSA: Bovine serum albumin
CDFDA: 5(6)-Carboxy-2′,7′-dichlorofluorescein diacetate
CLO: Cloxacillin
CYP: Cytochrome P450
DEGs: Differentially-expressed genes
DILI: Drug-induced liver injury
DMSO: Dimethyl sulfoxide
DPBS: Dulbecco’s phosphate buffer saline
ER: Endoplasmic reticulum
FBS: Foetal bovine serum
FLU: Flucloxacillin
GO: Gene ontology
GPX: Glutathione peroxidase
GSEA: Gene set enrichment analysis
GSR: Glutathione reductase
GST: Glutathione S-transferase
HO: Hoechst 33,342
HPLC: High performance liquid chromatography
KEGG: Kyoto Encyclopedia of Genes and Genomes
LDH: Lactate dehydrogenase
NAF: Nafcillin
PBS: Phosphate buffer saline
PCA: Principal component analysis
STR: Streptomycin
UGT: UDP-glucuronosyltransferase
