# Supplementary material for: A collagen hydrogel–based 3D Heparg model for enhanced hepatocyte function and assessment of cholestatic drug-induced liver injury
Source: Arch Toxicol. 2026 Mar 10;100(6):2559–80. doi: 10.1007/s00204-026-04324-z (PMC13221420; doi:10.1007/s00204-026-04324-z)
Supplement: Supplementary file 1 — Supplementary file1 (DOCX 1613 KB) [file 204_2026_4324_MOESM1_ESM.docx]

**SUPPLEMENTARY MATERIAL**

**Supplementary Figures**

**
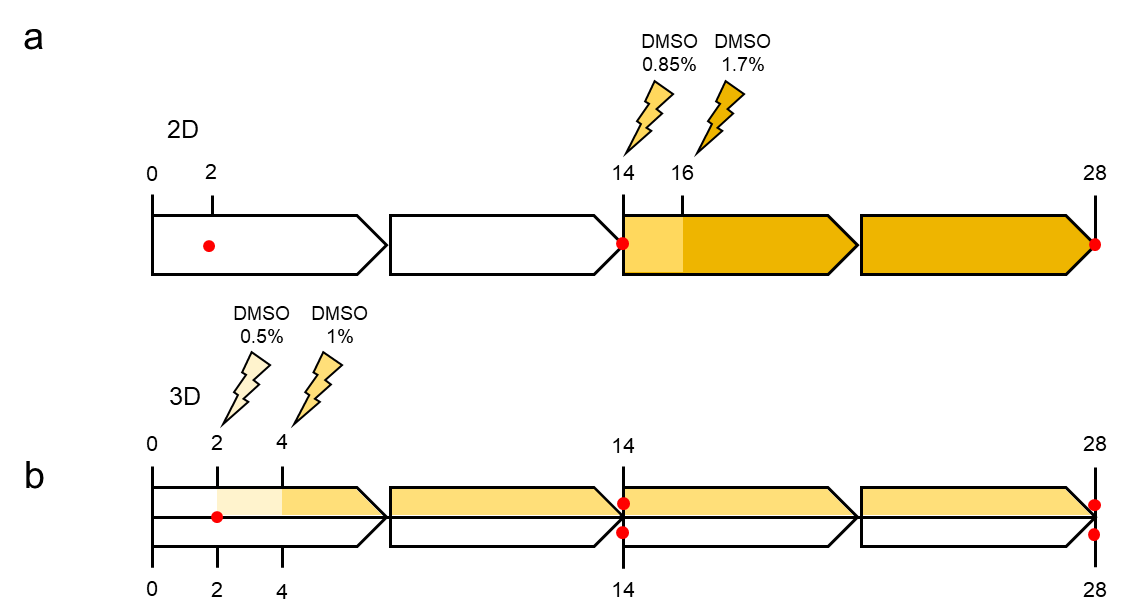
**

**Figure S1. Schedule of the experimental design.** HepaRG cells were cultured and differentiated in monolayers (a) or in 3D collagen hydrogels (b).

**
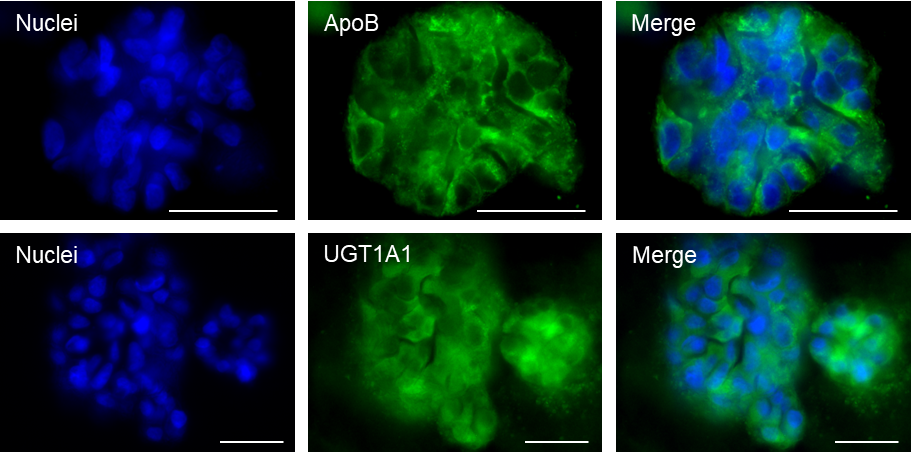
**

**Figure S2. Characterization of HepaRG cells cultured in 3D collagen hydrogels.** Representative immunofluorescence images of the ApoB and UGT1A1 (green) expression after 14 days of culture in collagen hydrogels with 1% DMSO. Nuclei were identified by Hoechst 33342 staining (blue). Scale bar (100 µm) applies to all the images.

**
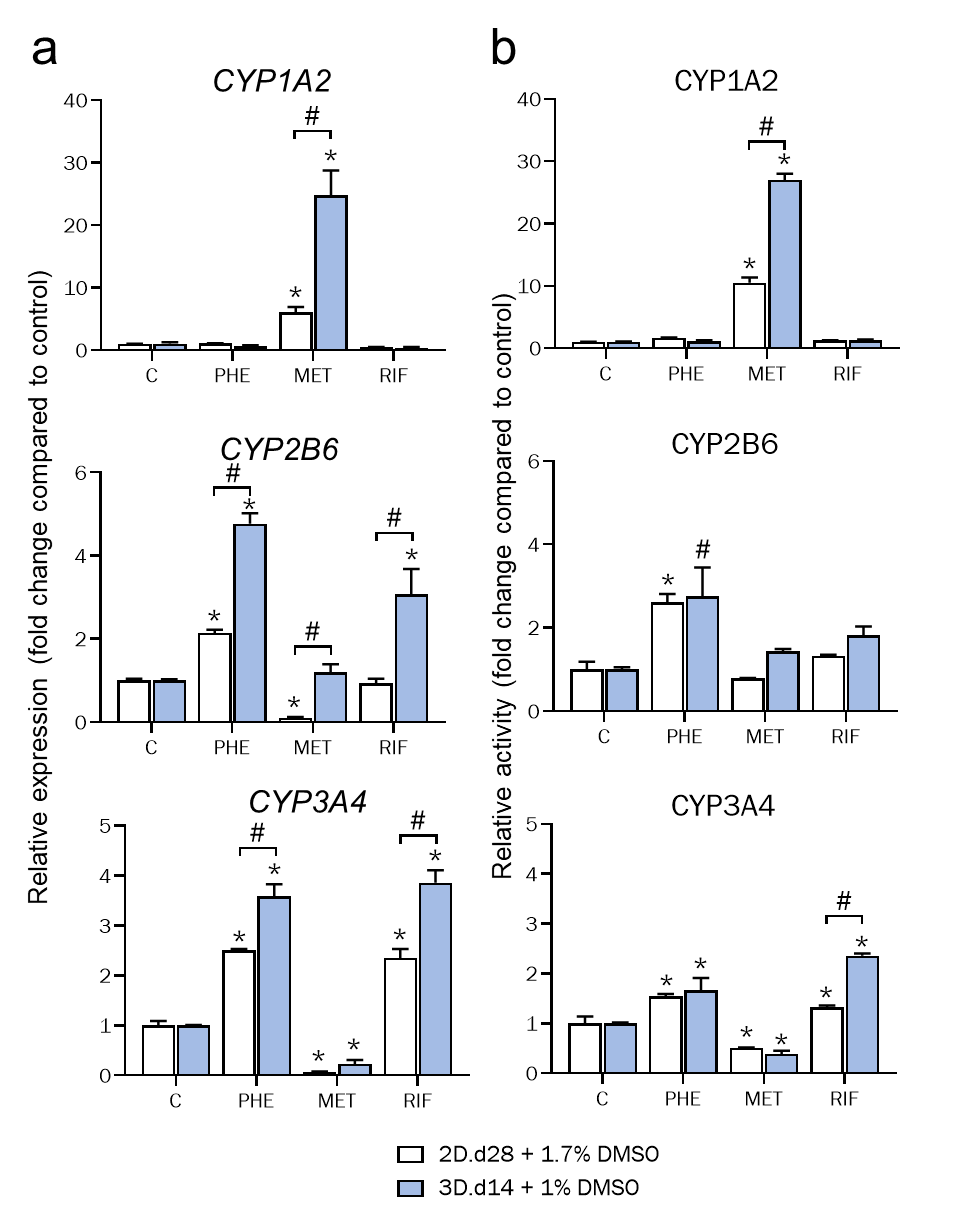
**

**Figure S3. Response to cytochrome P450 (CYP) inducers in HepaRG cells.** Gene expression (a) and enzymatic activity (b) of CYPs 1A2, 2B6, and 3A4 were studied in the monolayer (2D) model at 28 days in the presence of 1.7% dimethyl sulfoxide (DMSO) and in collagen hydrogels (3D) at 14 days in the presence of 1% DMSO after 48 h of induction with 1 mM phenobarbital (PB), 2 µM 3-methylcholanthrene (MET), and 25 µM rifampicin (RIF). Both types of measurements are expressed as relative change (FC) by normalizing each model to its untreated control. Statistics: two-way ANOVA with Dunnett's post-hoc multiple comparison test (each treatment vs. its control) or Student’s t test(2D vs. 3D); symbols indicate a p-value of at least <0.05; comparison of cells treated with each of the inducers vs. untreated cells (*) and between the 2D and 3D models for each treatment (#).

**
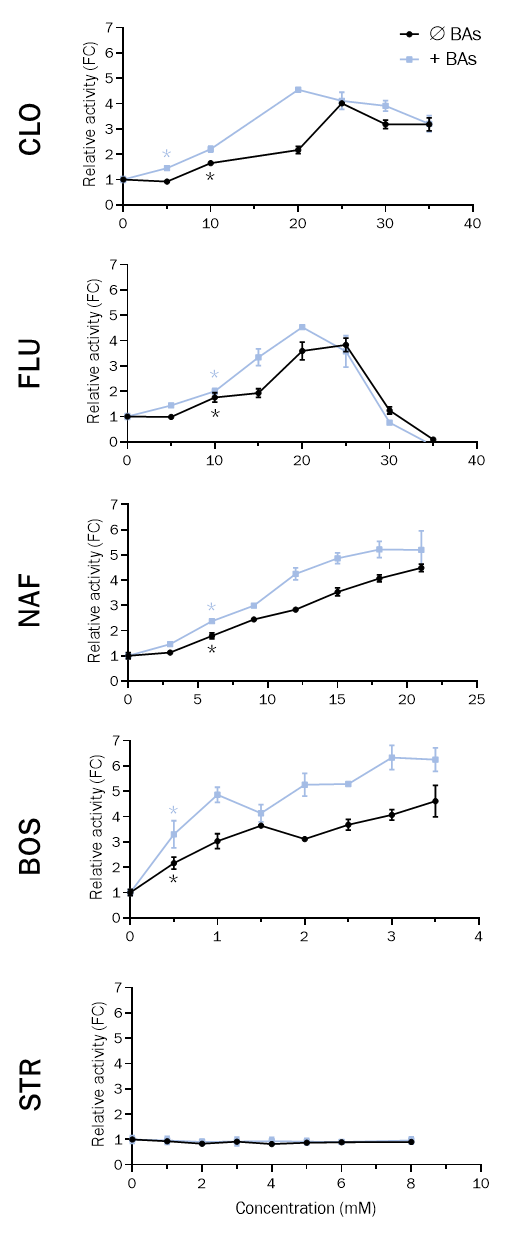
**

**Figure S4. Lactate dehydrogenase (LDH) enzyme activity in HepaRG cells after treatment with test compounds.** Statistics: two-way ANOVA with Dunnett's multiple comparisons post-test; the symbols (*) indicate the concentration from which the p value was at least < 0.05 when comparing viability at each concentration vs. the untreated control.

**
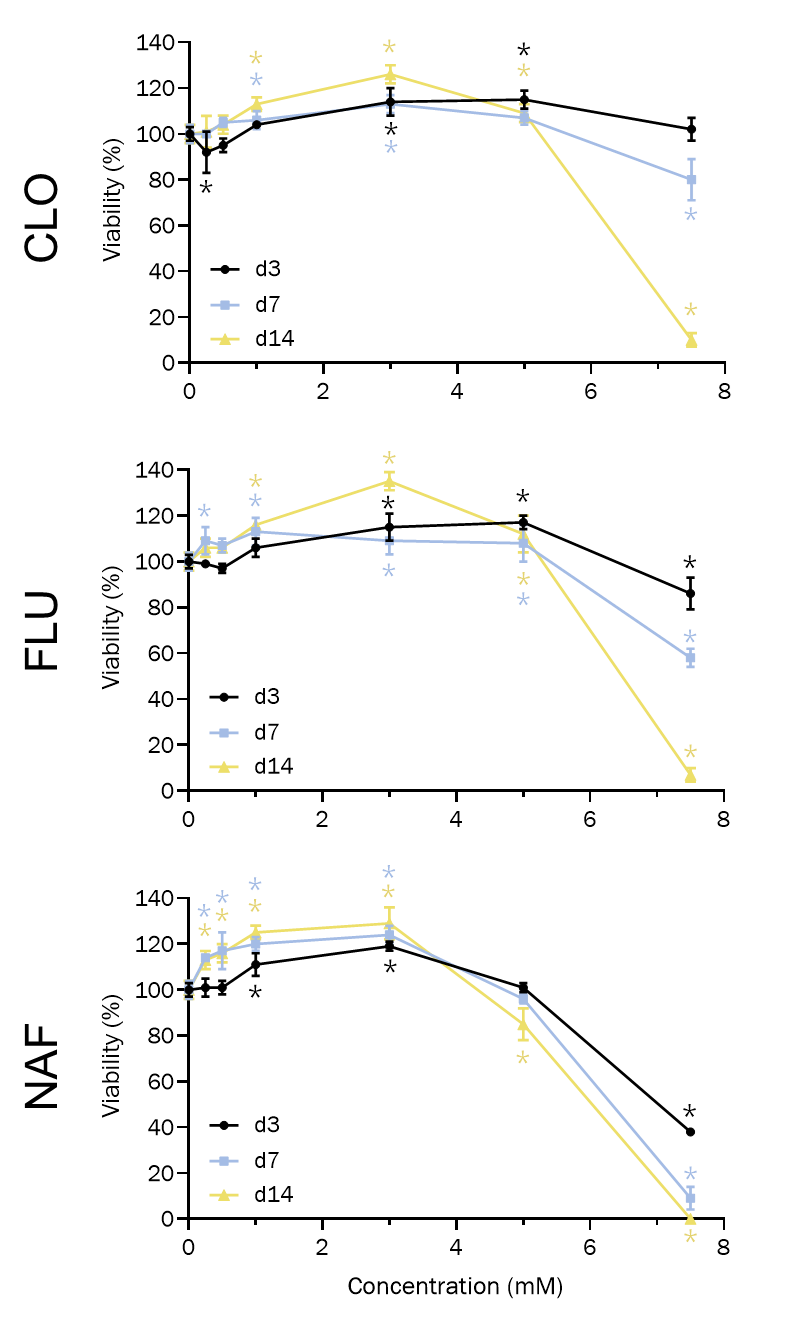
**

**Figure S5. Viability of HepaRG cells after exposure to different concentrations of cloxacillin (CLO), flucloxacillin (FLU), and nafcillin (NAF) for 3, 7, and 14 days.** Dose-response viability curves were generated based on intracellular ATP levels. *At least p< 0.05 when comparing viability at each concentration vs. untreated control (Two-way ANOVA with Dunnett's multiple comparisons post-test).

**
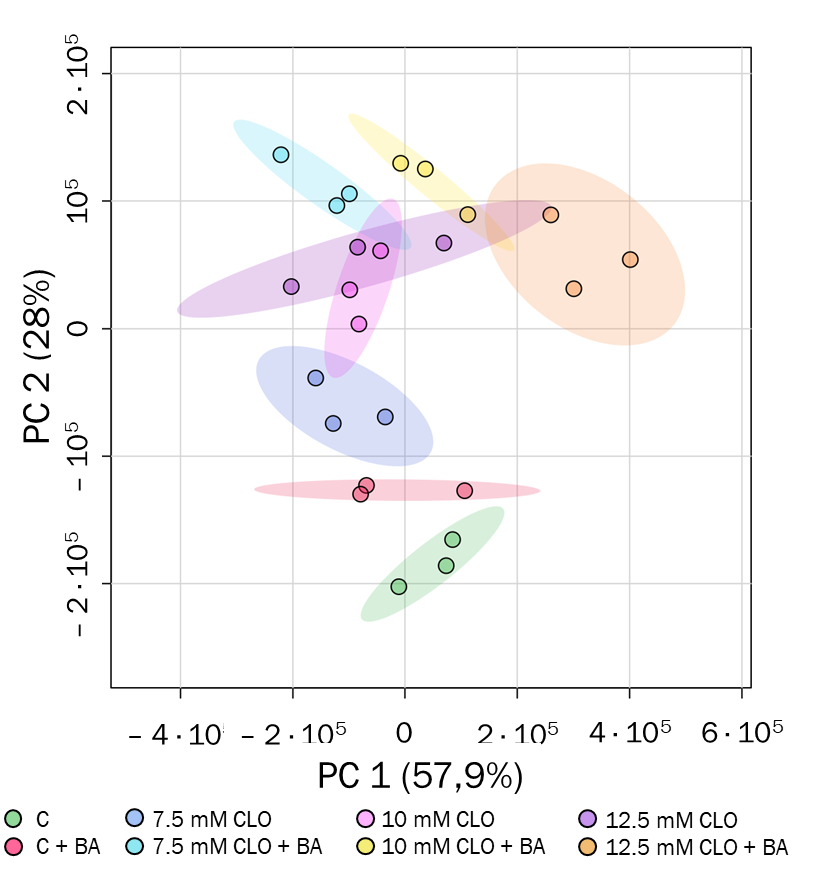
**

**Figure S6. Principal component analysis (PCA) of gene expression after treatment with cloxacillin (CLO).** RNAseq data from 3D HepaRG cells treated for 24 hours with three concentrations of CLO (7.5, 10, and 12.5 mM) in the absence and presence of bile acids (BA).

**
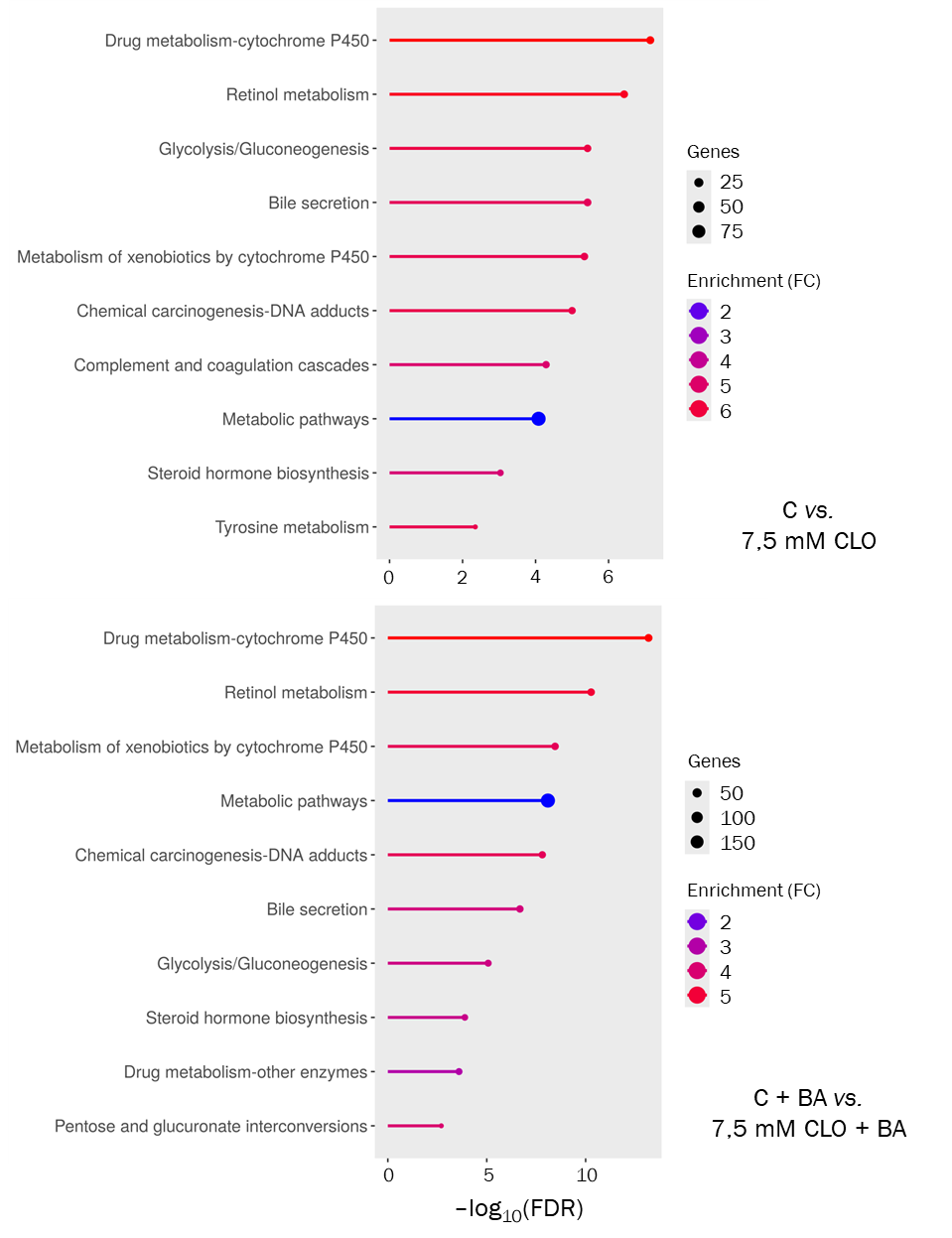
**

**Figure S7. Enrichment KEGG pathways.** Enrichment KEGG pathways of downregulated genes in cell treated with cloxacillin (CLO) 7,5 mM compared to control cells (C) and cells treated with the same concentration of cloxacillin in the presence of bile acids (BA), compared to cells culture in the presence of the BA cocktail**.** FC: fold change; FDR: false discovery rate.

**
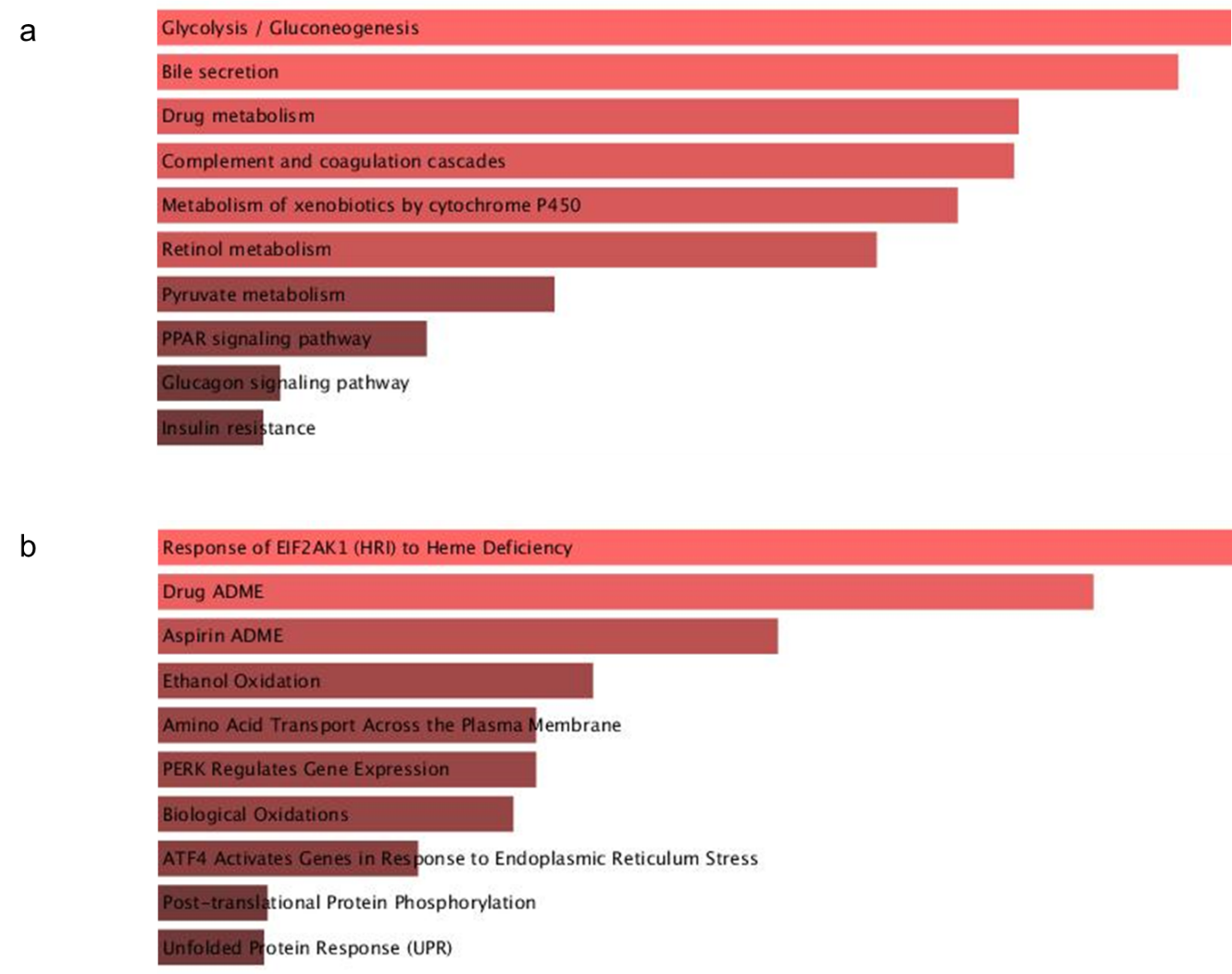
**

**Figure S8. Enrichment analysis of Transcriptional changes after cloxacillin treatment in 3D HepaRG cultures.** (**A**) KEGG analysis of overlapped genes altered after cloxacillin treatment. (**B**) Reactome Pathways of overlapped genes altered after cloxacillin treatment.

**
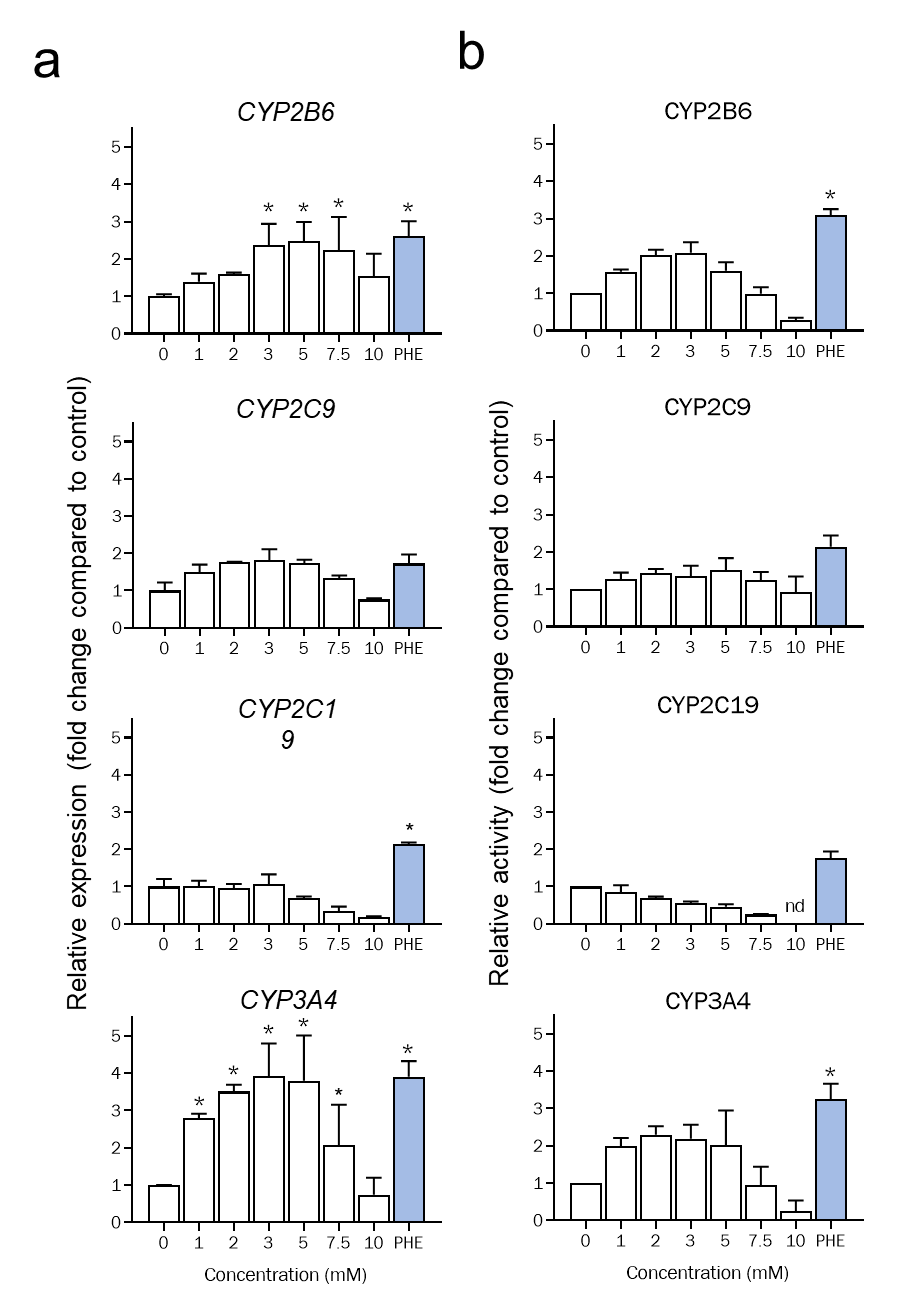
**

**Figure S9. Effect of cloxacillin (CLO) on gene expression (a) and enzymatic activity (b) of cytochrome P450 (CYP) enzymes in HepaRG cells.** Phenobarbital (PHE, 1 mM) was included as an inducer positive control. *At least p< 0.05 when comparing viability at each concentration vs. untreated control (one-way ANOVA with Dunnett’s multiple comparisons post-test).

**
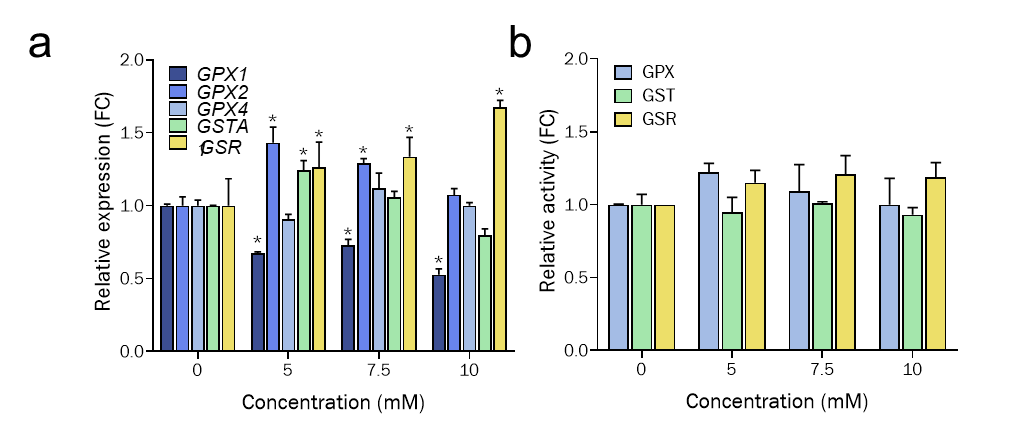
**

**Figure S10. Effect of cloxacillin (CLO) on gene expression (a) and enzymatic activity (b) of the enzymes glutathione peroxidase (GPX), glutathione S-transferase (GST), and glutathione reductase (GSR) in 3D HepaRG.** *At least p< 0.05 when comparing viability at each concentration vs. untreated control (one-way ANOVA with Dunnett’s multiple comparisons post-test).

**SUPPLEMENTARY TABLES**

**Supplementary Table S1**: The oligonucleotides used for quantitative RT-PCR

| **Symbol** | **Gene** | **Forward (5'->3')** | **Reverse (5'->3')** | **RefSeq.** | **nt position** |
| --- | --- | --- | --- | --- | --- |
| ***ABCB1*** | Multidrug resistance 1 | GGT TCT ACG ACC CCT TGG C | TGA AGG CAT GTA TGT TGG CCT | NM_001348945.2 | 3771-3989 |
| ***ABCB4*** | Multidrug resistance 3 | ACA GCC GGG TTG TAT CAC AG | GAT GAG GGC TCG GGC AAT AG | NM_000443.4 | 3457-3623 |
| ***ABCC2*** | Multidrug resistance-associated protein 2 | TCA GAC GAC CAT CCA AAA CGA | GGG TCC AGG GAT TTG TAG CAG | NM_000392.5 | 4630-4786 |
| ***ABCC3*** | Multidrug resistance-associated protein 3 | GGC TGA TGT TCC TGT GCT CC | ACC AGA GCC TTC CTG TAG ATG AC | NM_003786.4 | 1117-1240 |
| ***ABCC4*** | Multidrug resistance-associated protein 4 | GCA GTA CCT CAA AGC TGC AAG | CCG AAG ACT CTG AGA AGG TAC G | NM_005845.5 | 1919-2112 |
| ***ABCG2*** | ATP binding cassette subfamily G member 2 | CCA GGA CTC AAT GCA ACA GGA A | ACG TGA TTC TTC CAC AAG CCC | NM_004827.3 | 2050-2165 |
| ***ADH1A*** | Alcohol dehydrogenase | AAA CTT GTG GCT GAT TTT ATG GCT | GAC TGC CAC AAG GGA AAA CAT | NM_000667.4 | 1062-1230 |
| ***ALB*** | Albumin | CAC AGA ATC CTT GGT GAA CAG G | TGC GAA ATC ATC CAT AAC AGC | NM_000477.7 | 1544-1769 |
| ***ALDOB*** | Aldolase B | GAG GCT TTT ATG AAG CGG GC | TCC CTT TCA GCC CTC CTA CT | NM_000035.4 | 1052-1226 |
| ***APOB*** | Apolipoprotein B | GCC ACT TGG AGT GCC AGT TT | TGC ACT GAA GTC ACG GTG TG | NM_000384.3 | 12071-12274 |
| ***ARG1*** | Arginase | ACC TGC CCT TTG CTG ACA TC | CAG TGT GAG CAT CCA CCC AG | NM_001244438.2 | 236-463 |
| ***ASL*** | Argininosuccinate lyase | GGC GCG ACA CTA TCC GTG | GCA ATG GAC GCG TTG AAC TT | NM_001024943.2 | 8-349 |
| ***ASS1*** | Argininosuccinate synthase 1 | GCC TGG GCT TGA AAT TTG CT | GGT GGC ATC AGT TGG CTC AT | NM_000050.4 | 1291-1523 |
| ***ATF4*** | Activating transcription factor 4 | TGT GGA TGG GTT GGT CAG TC | AAC AGG GCA TCC AAG TCG AA | NM_001675.4 | 1076-1186 |
| ***ATF6*** | Activating transcription factor 6 | AAG TCC CTA GTC CAA AGC GAA G | GTG TCC TGT GCC TCT TTA GCA | NM_007348.4 | 1150-1327 |
| ***BAAT*** | Bile acid-amino acid N-aciltransferase | CCT CCT TGG CCT TGG CTT AC | GTA CCG TGG CTG TGA CTT GC | NM_001701.4 | 771-977 |
| ***CPS1*** | Carbamoyl-phosphate  synthetase 1 | GAG GTG GCT TGC TTT GGT GA | GGA CAT TGT TGG CGT TGA GC | NM_001122633.3 | 4097-4307 |
| ***CYP1A2*** | Cytochrome P450 1A2 | CCT TCG CTA CCT GCC TAA CC | CTC TAG GCC CCT TCT TGC TG | NM_000761.5 | 785-951 |
| ***CYP2B6*** | Cytochrome P450 2B6 | GTG TGG AGA AGC ACC GTG AAA C | GAG AGC GTG TTG AGG TTG AGG T | NM_000767.5 | 767-905 |
| ***CYP2C9*** | Cytochrome P450 2C9 | TCA AGA TTT TGA GCA GCC CC | AGT CAA CTG CAG TGT TTT CCA AG | NM_000771.4 | 639-905 |
| ***CYP2C19*** | Cytochrome P450 2C19 | ATG GAC ATC AAC AAC CCT CGG | CTC TGT CCC AGC TCC AAG TAA G | NM_000769.4 | 788-925 |
| ***CYP2D6*** | Cytochrome P450 2D6 | AAG TAC AGG GCT TCC GCA TCC | GGG CTC ACC AGG AAA GCA A | NM_000106.6 | 1167-1479 |
| ***CYP3A4*** | Cytochrome P450 3A4 | AGA AAG TCG CCT CGA AGA TAC AC | CAG AGC TTT GTG GGA CTC AGT T | NM_017460.6 | 874-973 |
| ***CYP7A1*** | Cholesterol 7α-hydroxylase | CGC AAG CAA ACA CCA TTC CA | AAC CGT CCT CAA GGT GCA AA | NM_000780.4 | 922-1197 |
| ***CYP7B1*** | Esterol 12α-hydroxylase | CCA TCC ACC TCA CCA GAG AAC | AAG TCT CCC TTT CGC ACA CA | NM_004820.5 | 1163-1325 |
| ***CYP8B1*** | 27-hydroxycholesterol  7α-hydroxylase | CTC TCC CCG CCT AGT CAT CT | GTT GCC CAC AGA TAC TCC CC | NM_004391.3 | 1717-2839 |
| ***CYP27A1*** | Esterol 27-hydroxylase | AAG GCT ATG CCC TGC AAC TG | CCA TGT CGT TCC GTA CTG GG | NM_000784.4 | 260-427 |
| ***DDIT3*** | C/EBP homologous protein | ATG AAC GGC TCA AGC AGG AA | GGG AAA GGT GGG TAG TGT GG | NM_001348945.2 | 750-897 |
| ***GAPDH*** | Glyceraldehyde 3-phosphate dehydrogenase | TTC GTC ATG GGT GTG AAC CA | GGC AGG GAT GAT GTT CTG GA | NM_002046.7 | 467-703 |
| ***GCLC*** | Glutamate-cysteine  ligase (catalytic subunit) | TCA ATG GGA AGG AAG GTG TGT | TGG TTT GCG ATA AAC TCC CTC A | NM_001498.4 | 2027-2207 |
| ***GCLM*** | Glutamate-cysteine  ligase (modifier subunit) | AAT CTT GCC TCC TGC TGT GTG | ACT CGT GCG CTT GAA TGT CAG | NM_002061.4 | 832-991 |
| ***GGT1*** | Gamma-glutamyl-  transferase 1 | GAC ACC TAC GAG ACG CTG G | ATC AGC TCA GCA CGG TAG TT | NM_001288833.2 | 1406-1545 |
| ***GPX1*** | Glutathione peroxidase 1 | CCC AAG CTC ATC ACC TGG TC | CGA TGT CAG GCT CGA TGT CA | NM_000581.4 | 514-652 |
| ***GPX2*** | Glutathione peroxidase 2 | GAC TTC ACC CAG CTC AAC GA | ATG CTC GTT CTG CCC ATT CA | NM_002083.4 | 155-358 |
| ***GPX4*** | Glutathione peroxidase 4 | CCA GTG AGG CAA GAC CGA AG | TCC ACT TGA TGG CAT TTC CCA G | NM_002085.5 | 263-540 |
| ***GSR*** | Glutathione reductase | ACA GTG GGA CTC ACG GAA GA | GTA GGG TGA ATG GCG ACT GT | NM_000637.5 | 1278-1549 |
| ***GSTA1*** | Glutathione S-transferase A1 | GTC GAG CCA GGA CGG TGA | CAT TCT GCC CCG TGC ATT GA | NM_145740.5 | 1-110 |
| ***HMBS*** | Hydroxymethylbilane synthase | ACC CTA GAA ACC CTG CCA GA | GAA GCC GGG TGT TGA GGT TT | NM_000190.4 | 550-676 |
| ***HNF4A*** | Hepatic nuclear factor 4α | TGC TGG TTC TCG TTG AGT GG | CAG CTC GTC AAG GAT GCG TA | NM_000457.6 | 718-950 |
| ***HSPA5*** | Immunoglobulin-binding protein | CAC AGT GGT GCC TAC CAA GA | TTT CTT CAG GTG TCA GGC GA | NM_005347.5 | 1537-1829 |
| ***JAG1*** | Jagged 1 | ACG TGA TGG AAA CAG CTC GC | GCC GTC ACC AAG CAA CAG AT | NM_000214.3 | 3592-3732 |
| ***KRT19*** | Keratin 19 | CTA GAG GTG AAG ATC CGC GAC | TGT CGA TCT GCA GGA CAA TCC | NM_002276.5 | 383-530 |
| ***MAT1A*** | Methionine adenosyltransferase 1A | AAG GAG CAA GTC ATC AGG GC | CCG CCA TAG GTG TCC ACA AT | NM_000429.3 | 902-1053 |
| ***MAT2A*** | Methionine adenosyltransferase 2A | CGG AGG GTT CTT GTT CAG GTC T | GCT GTC CCT ACC AAA GTG GC | NM_005911.6 | 1054-1272 |
| ***NR1H4*** | Farnesoid X-receptor | GAC GGA AAT GGC AAC CAA TCA | F-: R: GTC AGA ATG CCC AGA CGG AA | NM_001206979.2 | 1256-1433 |
| ***NR1I2*** | Pregnane X-receptor | TTG AAT GCA ATC GGC CCC AG | AGG GCA TTG TCG GCT CTT G | NM_022002.3 | 1295-1578 |
| ***OTC*** | Ornithine  transcarbamylase | GCT GAT TAC CTC ACG CTC CA | TGG TTA CAC TAG CAT CCG GC | NM_000531.6 | 613-784 |
| ***SERPINA1*** | Alfa-1-antitrypsin | GAA GAG CGT CCT GGG TCA AC | TGG TCA GCA CAG CCT TAT GC | NM_000295.5 | 1016-1137 |
| ***SLC10A1*** | Na^+^-taurocholate cotransporting polypeptide | CTG CTG GGT TAT GTT CTC TCT GC | CCA ATG ACT TCA GGT GGA AAG GC | NM_003049.4 | 2406-2514 |
| ***SLC22A1*** | Organic cation transporter 1 | ACC AGC GGG AAC CTC TAC CT | TGA TTC CCA TTC GGC CAA CA | NM_003057.3 | 1232-1448 |
| ***SLC51A*** | Organic solute transporter α | TGG GCA TCA TTT CCC GTC AAG | GTA GGG CAG TCA GGA TGA GGA | NM_152672.6 | 886-984 |
| ***SLCO1B3*** | Organic anion-transporting polypeptide 1B3 | GCA CTT GCA ATG GGT TTC CAG | TAA GCC CAA GTA GAC CCT TCC AAA | NM_019844.4 | 1945-2124 |
| ***SLCO2B1*** | Organic anion-transporting polypeptide 2B1 | GCC CTT TGG CAT CTC CTA CA | GGG GTC CTT TAT GGT CAG GC | NM_007256.5 | 896-1085 |
| ***SULT2A1*** | Sulfotransferase 2A1 | ATG AGT TCG TGA TAA GGG ATG | CTG AGT GCT GTA TAC CCA ATC T | NM_003167.4 | 141-321 |
| ***TBP*** | TATA-binding protein | TTC TGG GAT TGT ACC GCA GC | CGT GGT TCG TGG CTC TCT T | NM_003194.5 | 717-855 |
| ***TXNRD1*** | Thioredoxin reductase 1 | GGT GCT TGT GGC CTT TCT GA | GGA CCC AGT ACG TGA AAG CC | NM_182729.3 | 1365-1552 |
| ***UGT1A1*** | UDP-glucuronosyltransferase 1A1 | TGC GAC GTG GTT TAT TCC CC | AGG CTT CAA ATT CCT GGG ATA GTG | NM_000463.3 | 685-895 |
| ***UGT2B7*** | UDP-glucuronosyltransferase 2B7 | AGG AGC TAA ACA CCT TCG GG | TGC TGG AAT AAA CTG AAG TAG TCT C | NM_001074.4 | 1460-1699 |

**Table S2. List of antibodies used in this study.**

| **Immunofluorescence antibodies** | | | | | |
| --- | --- | --- | --- | --- | --- |
| **Target protein** | **Origin specie** | **Target species** | **Dilution** | **Brand** | **Ref.** |
| ***PRIMARY*** |  |  |  |  |  |
| A1AT | **R** | H | 1:100 | Merck | 223A-1 |
| Albumin | **G** | H, B, M, Pg | 1:100 | Bethyl | A80-229A |
| ApoB | **G** | H | 1:100 | Rockland | 600101111 |
| BSEP | **R** | H, M, Rt | 1:100 | Santa Cruz | sc-74500 |
| CYP3A4 | **M** | H, Rt | 1:100 | Santa Cruz | sc-53850 |
| HNF4α | **M** | H, M, Rt | 1:100 | Santa Cruz | sc-374229 |
| MDR1 | **M** | H, Hm | 1:100 | Sigma-  Aldrich | P7965 |
| MRP2 | **R** | H, M | 1:100 | Abcam | ab172630 |
| UGT1A1 | **R** | H, M, Rt | 1:100 | Proteintech | 23495-1-AP |
| ***SECONDARY*** |  |  |  |  |  |
| Goat-IgG  Alexa Fluor™ 488 | D | **G** | 1:200 | Invitrogen | A11055 |
| Goat-IgG  Alexa Fluor™ 594 | D | **G** | 1:200 | Invitrogen | A11058 |
| Mouse-IgG  Alexa Fluor™ 488 | D | **M** | 1:200 | Invitrogen | A32744 |
| Mouse-IgG  Alexa Fluor™ 594 | D | **M** | 1:200 | Invitrogen | A21202 |
| Rabbit-IgG  Alexa Fluor™ 488 | D | **R** | 1:200 | Invitrogen | A21206 |
| Rabbit-IgG  Alexa Fluor™ 594 | D | **R** | 1:200 | Invitrogen | A21207 |
| A1AT: alfa-1-antitrypsin; ApoB: apolipoprotein B; BSEP: bile salt export pump; CYP3A4: cytochrome P450 3A4; HNF4α: hepatic nuclear factor 4α; IgG: immunoglobulin G; MDR1: multidrug resistance protein 1; MRP2: multidrug resistance-associated protein 2; UGT1A1: UDP-glucuronosyltransferase 1A1. | | | | | |
| B: bovine; Ch: chicken; G: goat; Gp: guinea pig; H: human; Hm: hamster; Ho: horse; M: mouse; Pg: pig; R: rabbit; Rt: rat; Sh: sheep. | | | | | |

| **Western blot antibodies** | | | | | |
| --- | --- | --- | --- | --- | --- |
| **Target protein** | **Origin specie** | **Target species** | **Dilution** | **Brand** | **Ref.** |
| ***PRIMARY*** |  |  |  |  |  |
| β-tubulin | **R** | H, M, R, Mk, Z, B | 1:1000 | Cell Signaling | #2146 |
| AKT | **R** | H, M, R, Mk, Dm | 1:1000 | Cell Signaling | #4691 |
| p-AKT | **R** | H, M, R, Hm, Mk, Dm, Z, B | 1:2000 | Cell Signaling | #4060 |
| ERK1/2 | **M** | H, M, R, Hm, Mk, Mi, Z, B, Pg | 1:1000 | Cell Signaling | #9107 |
| p-ERK1/2 | **R** | H, M, R, Hm, Mk, Mi, Dm, Z, B, Dg, Pg, Sc | 1:2000 | Cell Signaling | #4370 |
| p38 | **R** | H, M, R, Hm, Mk, B, Pg | 1:1000 | Cell Signaling | #8690 |
| p-p38 | **R** | H, M, R, Mk, Mi, Pg, Sc | 1:1000 | Cell Signaling | #4511 |
| ***SECONDARY*** |  |  |  |  |  |
| Mouse-IgG HRP | G | **M** | 1:2000 | Dako | P0447 |
| Rabbit-IgG HRP | G | **R** | 1:2000 | Dako | P0448 |
| AKT: protein kinase B; ERK: extracelular signal-regulated kinase; HRP: horseradish peroxydase; IgG: immunoglobulin G. | | | | | |
| B: bovine; Dm: *Drosophila*; Dg: dog; G: goat; Gp: guinea pig; H: human; Hm: hamster; M: mouse; Mi: mink; Mk: monkey; Pg: pig; R: rabbit; Rt: rat; Sc: *Saccharomyces*; Z: zebrafish. | | | | | |

**y Table S2.** Immunofluorescence antibodies

**Table S3. BA mix used for evaluating the cholestatic potential.**

| **Bile acid** | **Concentration** | | | | |
| --- | --- | --- | --- | --- | --- |
|  | **Physiological [µM]*** | **Use (100X) [µM]** | **Cocktail [mM]** | | **Stock [mM]** |
|  | **–** | **1X** | | **250X** | **2000X** |
| Cholic (CA) | 0.20 | 20 | | 5.00 | 40 |
| Lithocholic (LCA) | 0.03 | 3 | | 0.75 | 6 |
| Deoxycholic (DCA) | 0.73 | 73 | | 18.25 | 146 |
| Chenodeoxycholic (CDCA) | 0.34 | 34 | | 8.50 | 68 |
| Ursodeoxycholic (UDCA) | 0.11 | 11 | | 2.75 | 22 |
| Glycocholic (GCA) | 0.41 | 41 | | 10.25 | 82 |
| Glycodeoxycholic (GDCA) | 0.38 | 38 | | 9.50 | 76 |
| Glycochenodeoxycholic (GCDCA) | 1.71 | 171 | | 42.75 | 342 |
| *Data from Scherer *et al.*, 2009. | | | | | |

**Table S4. Fluorescent probes used to determine mitochondrial-induced hepatotoxicity.**

| **Fluorescent Probe** | **Manufacturer** | **Reference** | **Final concentration (µM)** | **λ Excitation (nm)** | **λ Emission (nm)** |
| --- | --- | --- | --- | --- | --- |
| CDFDA | Sigma-Aldrich | 21884 | 5 | 470 | 529 |
| CellROX Deep Red | Molecular Probes | C-10422 | 5 | 644 | 665 |
| Fluo-4 AM | Molecular Probes | F-14217 | 0.25 | 494 | 516 |
| Hoechst 33342 | Sigma-Aldrich | B-2261 | 2.7 | 361 | 486 |
| MitoSOX Red | Molecular Probes | M-36008 | 5 | 510 | 580 |
| Propidium iodide | Sigma-Aldrich | P-4170 | 2.2 | 536 | 617 |
